# A high-throughput and low-waste viability assay for microbes

**DOI:** 10.1038/s41564-023-01513-9

**Published:** 2023-11-02

**Authors:** Christian T. Meyer, Grace K. Lynch, Dana F. Stamo, Eugene J. Miller, Anushree Chatterjee, Joel M. Kralj

**Affiliations:** 1 Molecular, Cellular, and Developmental Biology, University of Colorado Boulder, Boulder, CO USA; 2 Chemical and Biological Engineering, University of Colorado Boulder, Boulder, CO USA; 3 Antimicrobial Regeneration Consortium (ARC) Labs, Louisville, CO USA; 4 Duet Biosystems, Nashville, CO USA; 5 Sachi Bio, Louisville, CO USA; 6 Think Bioscience, Boulder, CO USA

**Keywords:** Microbiology, Molecular biology

## Abstract

Counting viable cells is a universal practice in microbiology. The colony-forming unit (CFU) assay has remained the gold standard to measure viability across disciplines, but it is time-intensive and resource-consuming. Here we describe the geometric viability assay (GVA) that replicates CFU measurements over 6 orders of magnitude while reducing over 10-fold the time and consumables required. GVA computes a sample’s viable cell count on the basis of the distribution of embedded colonies growing inside a pipette tip. GVA is compatible with Gram-positive and Gram-negative planktonic bacteria (*Escherichia coli, Pseudomonas aeruginosa* and *Bacillus subtilis*), biofilms and fungi (*Saccharomyces cerevisiae*). Laborious CFU experiments such as checkerboard assays, treatment time-courses and drug screens against slow-growing cells are simplified by GVA. The ease and low cost of GVA evinces that it can replace existing viability assays and enable viability measurements at previously impractical scales.

## Abstract (web summary)

Using agar media suspended in pipette tips and a standard camera, the geometric viability assay provides a user-friendly and scalable update to standard colony-forming-unit-count approaches.

## Main

The colony-forming unit (CFU) assay is the gold standard for enumerating viable cells in microbiology labs around the world^1–6^. The CFU assay combines simplicity with readily available reagents to achieve an enormous dynamic range, commonly measuring between 1 and 100,000,000 viable cells in a sample. Viability measurements are critical in numerous contexts spanning food safety^7^, functional genomics^8–10^ and drug discovery against persister cells^2,11^. However, measuring viability across numerous conditions using the CFU assay is time- and resource-intensive while generating a substantial amount of plastic waste^4,12^.

Previous approaches to increase the scale of viability measurements include (1) increasing the speed with robotic liquid handling and imaging^1,4,13^, (2) decreasing the amount of pipetting by using viability stains^14^ or microfluidics^15^ or (3) using cell growth to estimate the initial number of viable cells post-treatment^3^. The most commercially successful alternative to the CFU assay is the spiral plater method^16^ which deposits the sample in an Archimedes spiral on a solid medium plate. However, none of these approaches combines the simplicity, low cost, dynamic range and versatility of simply diluting cells and then growing drops on solid media as first proposed in ref. ^17^.

Here we developed a viability assay called the geometric viability assay (GVA). GVA calculates the CFUs in a sample on the basis of the axial position of embedded colonies that form in a cone. Intuitively, the probability of a colony forming at the tip of the cone is less than near the base due to differences in the cross-sectional area. Analytically, we find this probability to be proportional to the squared perpendicular distance of the colony to the cone tip. By measuring the position of a few colonies in the cone and utilizing the derived probability function, the total number of colonies in the entire cone can be computed with high precision. By leveraging the latent information encoded in the colony distribution, GVA accurately quantified the number of viable cells in a sample, ranging from 1 to 1,000,000 cells. This dynamic range was accomplished using a cone universal in microbiology—the pipette tip. In summary, GVA (1) measures viability over 6 orders of magnitude, (2) does not depend on the cell’s growth or lag phase, (3) minimizes consumables and (4) reduces operator time by over 30-fold compared with the drop CFU assay. Combined, this enabled a throughput of up to 1,200 viability measurements per researcher per day.

## Results

### The GVA

**Fig. 1:**
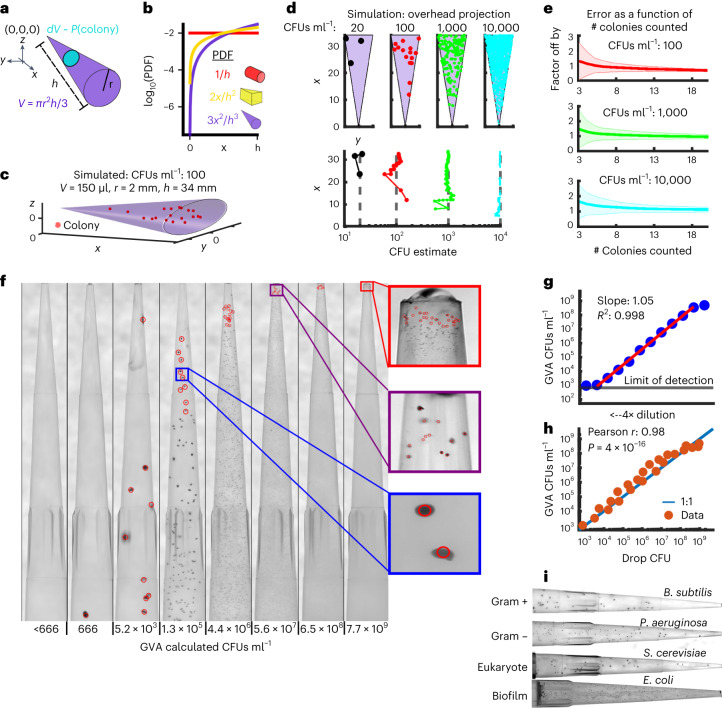
The GVA. **a**, The probability of a colony forming at a distance *x* from the tip of the cone is proportional to the infinitesimal volume *dV* (cyan circle) divided by the total volume *V* (purple cone). Analytically, this ratio is the probability density function (PDF) as a function of *x* (see Supplementary Information for derivation). **b**, The PDF for a cylinder (red), wedge (yellow) and cone (purple) as a function of the axial distance (*x*). **c**, Simulation of the colony distribution in a cone. **d**, Estimating the total CFUs ml^−1^ on the basis of the position of colonies in the cone. Top: distributions of colonies for 4 simulations spanning 20 to 10,000 CFUs ml^−1^ density. The volume of each cone is the same as in **c**. Bottom: GVA estimate of the CFUs ml^−1^ as a function of the included colonies and their *x* positions. **e**, The factor the GVA calculation differs from the correct value as a function of the number of colonies in equation (1). Solid lines and shaded error bars represent mean ± 1s.d. of 1,000 simulations. Colours match simulations in **d**. **f**, Dilution series of *E. coli* embedded in 150 µl 0.5% LB agarose in p200 pipette tips. Red circles correspond to colonies counted using custom semi-automated segmentation software. CFUs ml^−1^ estimates account for the initial 100× dilution of the sample into the agarose. **g**, *E. coli* CFUs ml^−1^ calculated using GVA for a 4× dilution series. Points are the mean of 4 replicates, calculated after taking the log. The red line is the linear regression fit to the dilution series. A slope of 1 on a log–log plot is expected if the GVA estimate scales linearly with dilution. **h**, The drop CFU and GVA estimates are significantly correlated over 6 orders of magnitude. Four technical replicates were used for each sample for both methods. Significance assessed using Pearson *r* correlation. **i**, GVA performed on planktonic Gram-positive and Gram-negative bacteria, eukaryotic cells and bacterial biofilms (see Extended Data Fig. 4a for quantification).

**Extended Data Fig. 1:**
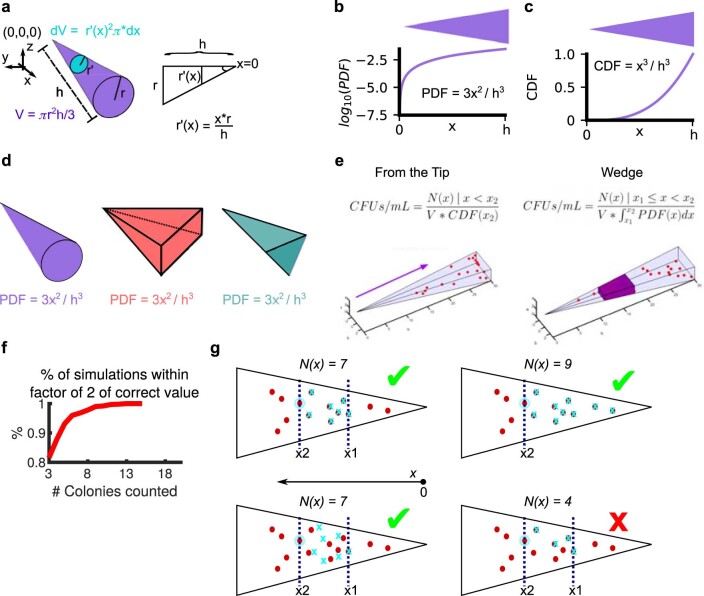
Derivation of a cone’s PDF. **a)** The volume of the infinitesimal *dV* divided by the total volume *V* corresponds to the probability of finding a colony as a function of *x*. The radius of the infinitesimal (*r'(x)*) is a function of the radius of the cone′s base (*r*) divided by the height of the cone (*h*) times *x* according to trigonometry. **b)** The PDF of the cone as a function of *x*. The overhead projection of the cone is depicted above. **c)** The cumulative density function (CDF) as a function of *x*. **d)** The PDF is the same for axially symmetric cones such as square (red) and triangle (turquoise) pyramids. **e)** Two equivalent ways of calculating the number of CFUs in the wedge using either the CDF (left) or PDF (right). *N(x)* is the number of colonies counted. **f)** Percentage of simulations with the GVA calculated CFUs/mL within a factor of 2 of the correct value as a function of the number of colonies used for the GVA calculation. 1000 simulations were used to calculate the percentage. See Fig. 1c for simulation parameters. **g)** Examples of counting colonies in GVA. Red dots correspond to colonies. Cyan **x**’s are counted. Open circles are not counted, but their position is required for the integral. The left 2 panels are equivalent. The upper right panel is also correct but uses the CDF equation. The bottom right panel is incorrect as not all colonies were counted between x1 and x2.

The most time- and resource-intensive step of the classic drop CFU method is the dilution series that must be run to count individual colonies across several orders of magnitude. We reasoned that the geometry of a cone could create a dilution series in a single step as the cross section at the tip is less than the cross section near the base. Analytically, the probability of a colony forming at any point along the cone’s axis is proportional to the cross-sectional area at that point (Fig. 1a, cyan circle). This probability is defined as the probability density function (PDF) equal to

1 $${\rm{PDF}}\left(x\right)=\frac{3{x}^{2}}{{h}^{3}}$$

where *x* is the perpendicular distance from the tip along the *x* axis and *h* is the total length of the cone (Fig. 1a and Extended Data Fig. 1a–c; see Supplementary Information for derivation). Equation (1) is applicable for arbitrary cones or pyramids that are axially symmetric (Extended Data Fig. 1d). The total CFU concentration in the cone can be estimated as

2 $${\rm{CFUs}/{mL}}=\frac{\left(N\left(x\right) | {x}_{1}\le x < {x}_{2}\right)}{V {\int }_{{x}_{1}}^{{x}_{2}}{\rm{PDF}}\left(x\right){dx}}$$

where (*x*_1_,*x*_2_) are the positions of the first and last colony in the counted sub-volume and *V* is the volume of the cone. Thus, the highest CFU density resolvable is proportional to the dynamic range of the PDF. In contrast to a cylinder or a wedge, the cone achieves the maximum dynamic range in the PDF by changing shape in all 3 dimensions (Fig. 1b). Importantly, this probability does not depend on the radial (*y*,*z*) position of a colony within the cone, only on the perpendicular distance from the tip along the cone’s axis (*x*).

We simulated colony distributions in a cone for different CFUs ml^−1^ (Fig. 1c,d). As expected, the more CFUs in the cone, the more colonies are found near the tip (Fig. 1d, top). The CFUs ml^−1^ estimate quickly converges to the correct value (grey dotted line) as more colonies’ positions are included in equation (2), regardless of the colony density (Fig. 1d, bottom). Remarkably, the CFU estimate is off by less than a factor of 2 from the correct value in 97% of simulations based only on the positions of the first 10 colonies, even if there are over 10,000 colonies in the cone (Fig. 1e and Extended Data Fig. 1f). This rapid convergence to the correct value is the same regardless of the CFU concentration. Therefore, by leveraging the information encoded in the geometry of the cone, we can accurately calculate the colony density without necessarily counting all the colonies. This concept is analogous to a three-dimensional (3D) haemocytometer; by counting a subset of colonies within a defined volume, the total concentration can be computed using probabilities.

### Microbial viability testing inside a pipette tip

**Extended Data Fig. 2:**
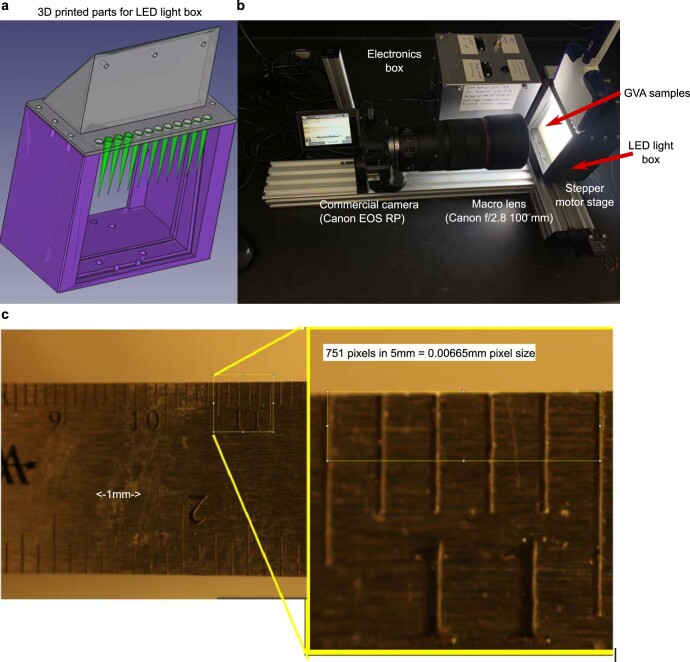
Optical configuration. **a)** CAD model showing the 3D printed optical box with lid (gray) holding pipette tips (green) attached to the base (purple). The base holds the glass plate, diffusion plate, and the LEDs used to illuminate the pipette tips in *trans*. **b)** Picture of optical configuration. The camera is mounted on a rail system perpendicular to a stepper motor rail with the optical box mounted. The GVA samples are positioned using a 12-channel pipetter held in position with the optical box lid and imaged using a Canon EOS RP camera with an f/2.8 100 mm macro lens. The electronics box houses the circuitry required to control the LEDs and stepper motor. An Arduino Uno is used to control both which interfaces with the MATLAB application used to segment the pipette tip images (see Methods). **c)** Pixel resolution for this configuration is 6.65 microns.

To test the theory, we used a cone ubiquitous in microbiology—the pipette tip. The first experiment was a dilution series using stationary-phase *Escherichia coli* (BW25113). Stationary-phase *E. coli* are known to have ~10^9^ CFUs ml^−1^ after overnight growth^18^. Stationary-phase cells were serially diluted and then each dilution was treated as a sample of unknown concentration of viable cells. Each ‘sample’ was fully mixed with melted LB agarose (cooled to ≤55 °C) to a final agarose concentration of 0.5%. Triphenyl tetrazolium chloride (TTC) was included in the melted agarose to increase the colony contrast. The agarose was allowed to solidify in the tip before the tip was ejected into an empty tip rack (see Methods). The agarose-containing pipette tips were then incubated overnight at 37 °C and imaged the following day using a custom-built optical setup with a mirrorless Canon camera (Fig. 1f; see Extended Data Fig. 2 for optical configuration and Supplementary Movie 1 for GVA protocol overview). See Methods for the parts list, circuit design and fabrication instructions for the GVA optical system. In agreement with our simulations, the distribution of colonies that form in the tip was predictable based on the PDF across >6 orders of magnitude (Fig. 1g, slope ~1). Remarkably, the final colony size decreased with increasing colony density, which prevented colony overlap even at high densities.

**Extended Data Fig. 3:**
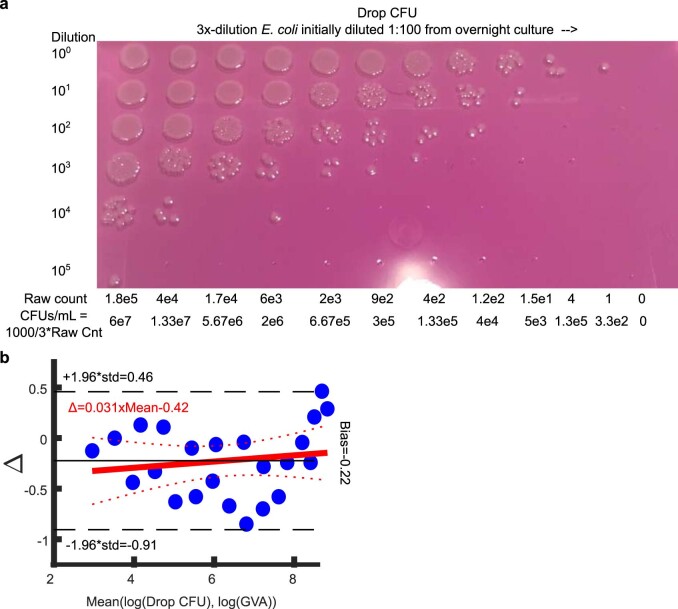
Example drop CFU plate and Bland-Altman analysis. **a)** Each sample (columns) is diluted with a 10-fold serial dilution (rows) and 3 µL are spotted on a 1.5% LB agar pad. Colonies are counted for the dilution row where individual colonies are discrete. These counts are used to calculate the CFUs/mL (bottom). **b)** Bland-Altman plot comparing GVA and drop CFU measurements. CFU values were log-transformed for the comparison. The method difference (Δ) as a function of the mean of the methods (x-axis) was fit using a linear regression model (red line, equation) with confidence intervals depicted (dotted red lines). The limits of agreement, equal to 1.96X the standard deviation in Δ between the methods is depicted in the black dotted lines. The bias, equal to the mean Δ across the data is annotated in the solid black line.

To compare GVA-estimated colony counts to ground-truth CFU counts, the CFUs ml^−1^ were measured using the drop CFU method (see Methods and Extended Data Fig. 3a) for 24 different dilution samples of overnight *E. coli* cells ranging up to a 4 × 10^6^-fold dilution. Each ‘sample’ was tested independently in technical quadruplicate using both drop CFU and GVA. Comparison of the calculated CFUs ml^−1^ using GVA and the traditional drop CFU assay showed that the two approaches are significantly correlated (Fig. 1h, Pearson *r* = 0.98, *P* = 4 × 10^−16^). We further examined the agreement between drop CFU and GVA using Bland–Altman analysis (Extended Data Fig. 3b). GVA had an average bias of less than a factor of 2 (Bias = 10^−0.22^ = 1.6) across 6 orders of magnitude. The trendline slope of the method difference (Δ) as a function of the CFUs ml^−1^ was statistically indistinguishable from zero (*F*-statistic vs constant model: 0.524, *P* = 0.477).

### GVA is applicable in both model and environmental samples

**Extended Data Fig. 4:**
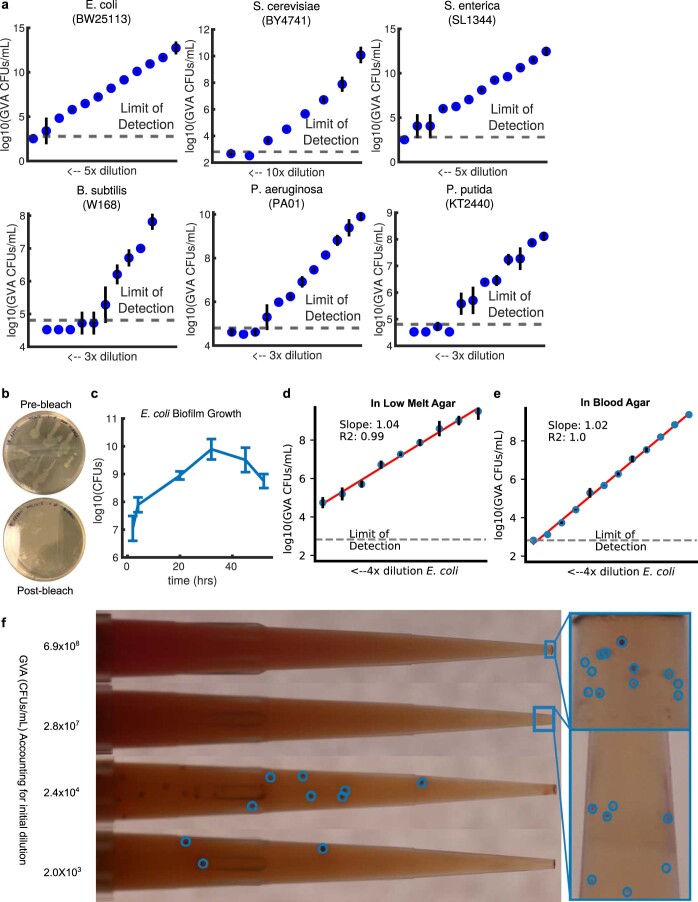
GVA calculations for different species. **a)** For the six species tested with GVA, the average number of CFUs/mL between 3 biological replicates for different dilution series. Error bars represent the standard deviation between replicates. **b)** Plates streaked with pipette tip after GVA embedding before or after bleach wash. No change in CFUs/mL was observed after a bleach wash. **c)** Biofilm growth over time. Error bars correspond to the standard deviation between 6 biological replicates and points represent the mean. **d)** Mean GVA using Low Melt Agar quantifying a dilution series of *E. coli* cells between 3 biological replicates. Error bars represent the standard deviation between replicates. **e)** GVA in blood agar (5% sheeps blood) with *E. coli* cells. **f)** Example tips from with blood agar showing *E. coli* colonies. Colonies stained with TTC.

GVA was used to count other Gram-negative (*Pseudomonas aeruginosa*, *Salmonella enterica* serovar Typhimurium, *Pseudomonas putida*) and a Gram-positive bacterial strain (*Bacillus subtilis*) as well as eukaryotic yeast cells (*Saccharomyces cerevisiae*) (Fig. 1i and Extended Data Fig. 4a). Enclosing the colonies in a pipette tip facilitated handling of pathogenic strains because a bleach wash could kill all contaminating cells on the outside of the tip without affecting colony growth inside the tip (Extended Data Fig. 4b). Viability in *E. coli* biofilms over time was also tested with GVA (Fig. 1i and Extended Data Fig. 4c).

**Extended Data Fig. 5:**
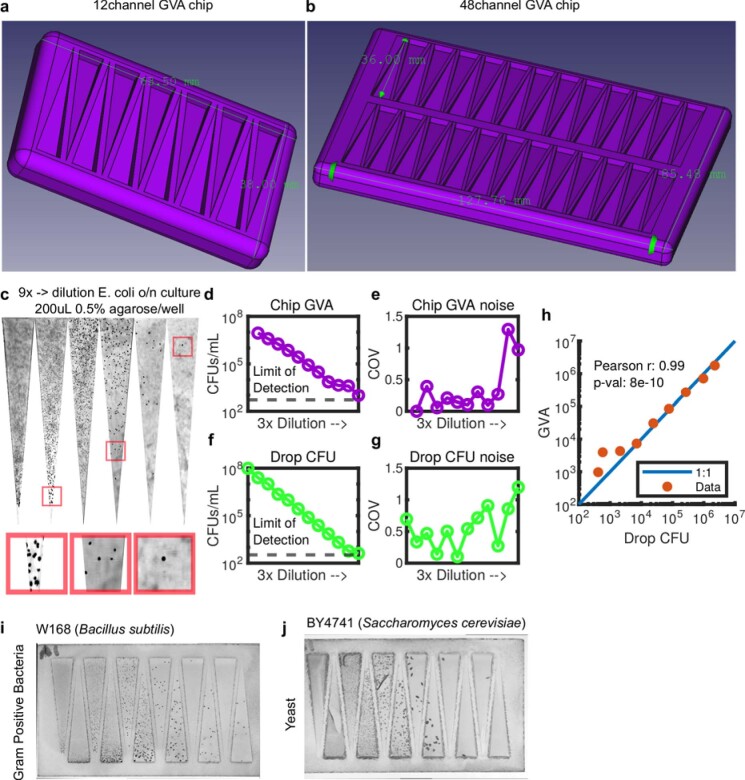
Square pyramid version of GVA. **a, b)** 3D printed molds for creating square pyramid for 12 (a) and 48 (b) conditions. **c)** Picture of a 9x dilution series of *E. coli* cultures on the GVA chip. **d)** GVA calculated CFUs/mL using for a dilution series. Each dot is the mean of 4 technical replicates. **e)** The noise is measured using the coefficient of variation (COV) for the chip GVA. **f)** Matched drop CFU quantification to conditions in (d). **g)** Corresponding noise analysis for drop CFU. The error bars represent the standard deviation between 4 technical replicates. **h)** Correlation between chip GVA and drop CFU over 5 orders of magnitude. The error bars represent the standard deviation between 4 technical replicates. Significance assessed using Pearson correlation. **i, j)** Chip GVA for Gram-positive (i) and eukaryotic (j) cells.

For all cultures, samples were embedded in 0.5% agarose melted in culture medium: LB for the bacteria and YEPD for the yeast. Low-melt agarose cooled to 37 °C before embedding and then incubated at room temperature was used for temperature-sensitive samples (Extended Data Fig. 4d). Embedded bacterial colonies also grew in other media such as Mueller–Hinton broth or M9 minimal medium and were even discernible in blood agar despite its low transparency and red coloration (Extended Data Fig. 4e,f). Using 3D-printed moulds to embed the colonies in a square pyramid (Extended Data Fig. 5a,b), we confirmed that the GVA approach applied to geometries other than circular cones, as predicted (Extended Data Figs. 1d and 5c–j).

**Extended Data Fig. 6:**
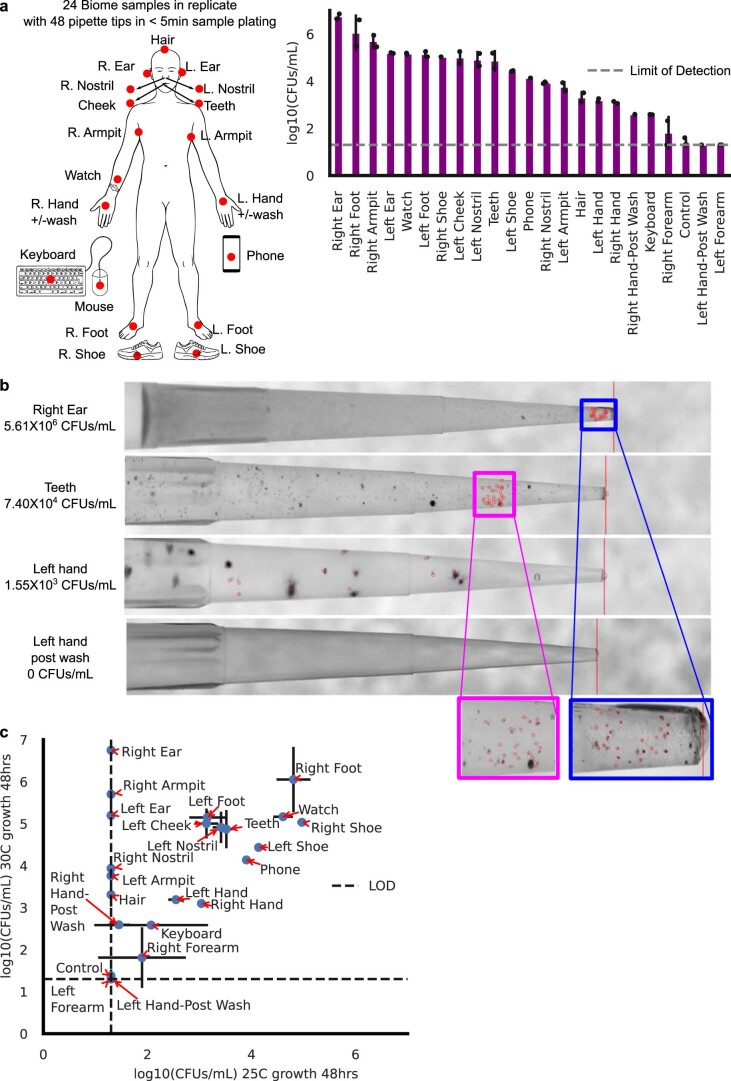
Biome sampling using GVA. **a)** Twenty-four positions (red dots) on a volunteer were swabbed vigorously for 15 seconds before being placed in 1 mL of LB medium and vortexed for 10 seconds. 50 µL of the sample was then mixed with 150 µL of 0.66% melted LB agar to a final concentration of 0.5% agar and allowed to gel in the tips. With this protocol, the lower limit of detection was 20 CFUs/mL (dotted line). The sample replicates were incubated at 30 °C or 25 °C for 48 hours before imaging. Error bars denote the standard deviation between 3 biological replicates and bars the mean. **b)** Example pipette tips for different sample regions reveals diverse colony structure and concentration for different biome locations. All samples were stained with TTC. **c)** Samples from higher thermal regions (ear, armpit) grew at 30 °C but did not grow at 25 °C indicating the temperature selectivity of different species grown in the pipette tip.

The potential of GVA for rapid quantitation of non-model bacterial species was also tested. Human-associated biome viability measurements were conducted using GVA (Extended Data Fig. 6). Vigorously swabbing 24 locations (Extended Data Fig. 6a) revealed a large dynamic range of microbial concentrations capable of aerobic growth in LB (Extended Data Fig. 6b). Growing sample replicates at different temperatures revealed temperature-selective growth for different biomes (Extended Data Fig. 6c). These experiments necessarily underestimate the number of bacteria in these biomes as many human commensals are unculturable. However, because GVA uses solid growth media, the same selective culturing techniques developed over the past 100 yr for standard Petri dish plating can be leveraged in GVA while also enabling high-throughput surveillance of culturable biomes.

### GVA dependence on optical resolution

**Fig. 2:**
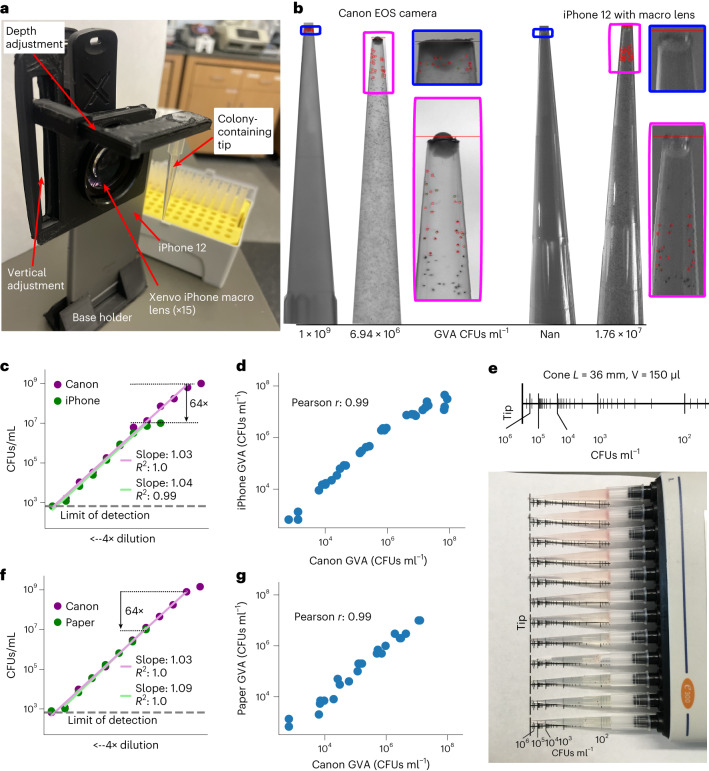
GVA dynamic range, but not accuracy, depends on the optical configuration. **a**, Picture of assembled pipette tip holder on an iPhone 12 with a Xenvo macro lens. The pipette images were taken in front of a white backdrop (paper) with ambient illumination. **b**, Example images of the same 2 pipette tips using the Canon EOS with 100 mm f2.8 macro lens (left) or the iPhone 12 with Xenvo macro lens (right). The GVA-calculated CFUs ml^−1^ are reported at the base of the tips. Selected colonies for GVA calculation are boxed. **c**, Dynamic range of the iPhone GVA. *E. coli* were diluted 4× and embedded in pipette tips. After incubation, the same tips were imaged with the iPhone camera with a macro lens (green) and the mirrorless camera (purple). Points are the average of 4 replicates calculated after taking the log. The green and purple lines are the linear regression fits to the dilution series. **d**, Pearson correlation between iPhone GVA and Canon camera for all pipettes where colonies could be counted using both. The correlation coefficient was calculated in log space. **e**, Top: ruler annotating the expected position of the 10th colony from the tip of a 36 mm, 150 µl cone for different concentrations of CFUs ml^−1^. Bottom: paper-based GVA. **f**,**g**, Dynamic range (**f**) and correlation (**g**) of paper-based GVA measurements (green) to the Canon camera (purple). Points are the mean of 4 technical replicates.

**Extended Data Fig. 7:**
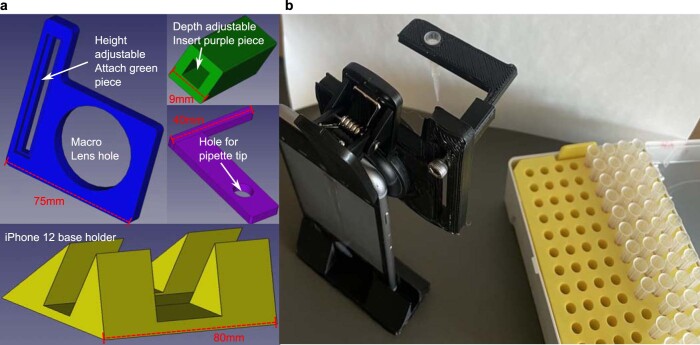
iPhone pipette tip holder. **a)** The 3D printed parts for stereotypically positioning a pipette tip in front of an iPhone rear camera with a Xenvo macro lens (15x magnification without the widefield lens). The blue face plate slides onto the Xenvo macro lens which is clipped to the iPhone. The green bar is attached with a screw to the side channel on the blue plate. This allows for adjusting the height by sliding the green bar in the channel. The purple extension bar slides into the green channel to adjust the imaging depth. **b**) The phone is held upright with a stand (yellow). Pieces printed with standard FDM printing with PLA.

We next investigated how the dynamic range and accuracy of GVA depended on the optical configuration using a low-cost camera system—an iPhone 12 with a commercial macro lens. We designed a pipette tip holder that positioned a single tip in front of an iPhone 12 rear camera and a macro lens (Fig. 2a and Extended Data Fig. 7). Calibration revealed the pixel size of the iPhone 12 to be 13.7 µm compared with 6.6 µm for the Canon EOS camera with 100 mm f/2.8 macro lens (Extended Data Fig. 2). We reasoned that the larger pixel size and lower electron depth in the iPhone 12 camera would reduce the smallest possible colony detected as compared with the mirrorless camera. As expected, comparing images taken with the Canon camera with those taken with the iPhone 12 demonstrated that colonies at the highest CFU concentrations were no longer resolvable on the iPhone (Fig. 2b). Comparing the GVA-calculated CFUs ml^−1^ for the same pipette tips of an *E. coli* dilution series using both the iPhone 12 and the Canon camera, we measured a reduction in the dynamic range of 64× on the iPhone 12 as compared with the Canon camera (Fig. 2c). However, GVA remained highly linear for nearly 5 orders of magnitude (green line, slope = 1.04, *R*^2^ = 0.99) with the iPhone 12 configuration. The Pearson correlation between the CFU counts for the iPhone and Canon configurations on the same pipette tips was 0.99 (Fig. 2d).

We reasoned that we could bypass the need for a camera by encoding the mathematics of GVA on a piece of paper in the form of a ruler (Fig. 2e). Converting equation (2) to use the cumulative probability density function (see Supplementary Information for derivation), the ruler annotates the expected *K*^th^ colony from the tip for a given cone geometry and concentration of CFUs. By printing this ruler on a piece of paper, aligning the pipette tips along the ruler and using a handheld ×30 magnifying glass to see the smallest colonies, we achieved the same performance as with the iPhone (Fig. 2f,g). Software for generating these rulers for arbitrary geometries is included in Methods.

Therefore, we found that GVA is accurate regardless of the optical configuration, but the dynamic range is set by the maximum colony resolution.

### Cost and time savings of GVA

**Fig. 3:**
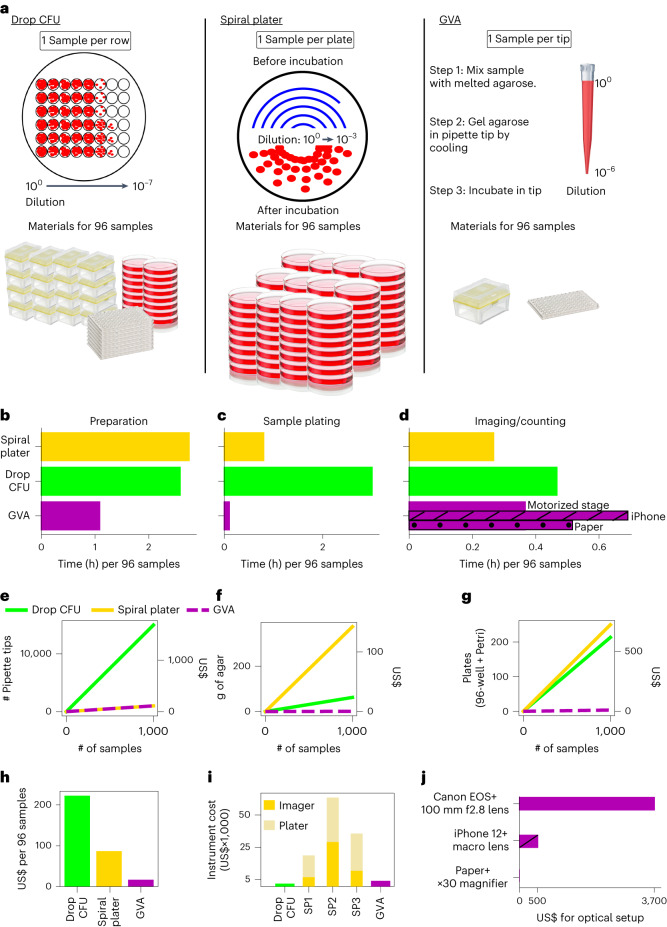
GVA reduces the time and materials for viability measurements by over 10-fold. **a**, Left: schematic of a drop CFU assay and required materials for 96 samples assuming tips are changed for each dilution step. Middle: a spiral plater spreads a sample in an Archimedes spiral on a solid media plate. The spiral results in decreasing sample volume as a function of radial distance with a reported 3-log dynamic range. One Petri dish is required per sample. Right: GVA uses a single pipette tip to achieve a 6-order dynamic range. **b**–**d**, Time comparisons for different techniques. **b**, Time required to prepare solid growth media. **c**, Sample plating from a 96-well plate. **d**, Time required for quantification of 96 samples. **e**, Number and cost of pipette tips as a function of sample count for the three different techniques. See Supplementary Table 1 for cost estimates. **f**, Amount of agar required as a function of sample count. **g**, Number of 96-well plates and Petri dishes per condition. **h**, Estimated total cost in consumables per 96 samples using the three methods. GVA cost is US$0.17 per sample. **i**, Instrument costs. SP, spiral plater. **j**, The differences in instrumentation costs for the Canon, iPhone 12 and paper-based optical configurations.

The main advantage of GVA is the >10× reduction in time, reagent cost and plastic waste as compared with the drop CFU and spiral plater methods (Fig. 3). The spiral plater is the most common commercial alternative for the CFU assay, utilizing a specialized instrument to dilute the sample along an Archimedes spiral^16^. To measure the time savings of GVA, we compared 3 steps of viability assays including: the preparation of solid growth media (Fig. 3b), diluting/plating 96 conditions (Fig. 3c) and imaging/counting of the colonies (Fig. 3d). The largest time savings was in the plating step. The drop CFU took 3 h to manually plate 96 conditions. Current spiral plater instruments are reported to take 30 s per plate, corresponding to 96 conditions in 48 min. GVA took 5 min, corresponding to a 36× and 9× savings in time for plating over the drop CFU and the spiral plater approaches, respectively. GVA was also faster in terms of preparation time than both the spiral plater and drop CFU approaches. The time for imaging and counting of the colonies was fastest on the spiral plater according to the manufacturer-reported time using an automated colony counter. GVA semi-automated colony counting took a similar amount of time to manual colony counting for the drop CFU when including the time for image acquisition, pipette tip segmentation and user-guided colony detection. In total, using the current instrumentation, a single researcher measured the viability of 1,200 conditions in a day.

We next compared the reagent savings and plastic waste reduction of the three approaches. In the drop CFU assay, since each sample must be diluted and then separately transferred to an agarose pad, 15 pipette tips per sample are standard for our laboratory protocol (Fig. 3e)^19^. In GVA, a single pipette tip is used per sample, amounting to a 15× savings in pipette tips over the drop CFU (Fig. 3e). In the spiral plater assay, a Petri dish with a solid growth medium is required per sample (Fig. 3g). Compared with the spiral plater method, the plastic required is reduced from a Petri dish to a pipette tip. Summing the cost of pipette tips, agar and culture plates at the time of writing, we found the drop CFU to be the most expensive in consumables, costing an average of US$222 per 96 samples compared with the spiral plater and GVA which cost US$87 and US$17, respectively (Fig. 3h and Supplementary Table 1 for pricing rationale). The savings in consumables of the spiral plater are offset by the substantial instrument costs (Fig. 3i and Supplementary Table 1). Costs were calculated from quotes for 3 spiral platers and automated imaging systems solicited from three distributors. The instrument costs for both the GVA and the drop CFU included a multichannel pipette. Additional instrumentation costs for the GVA depended on the optical configuration (Fig. 3j), which were an order of magnitude less than the spiral plater systems.

In summary, our analysis showed that GVA substantially reduced operator time, instrument and reagent costs, and the carbon footprint of viability assays.

### Robustness and noise of GVA experiments

**Fig. 4:**
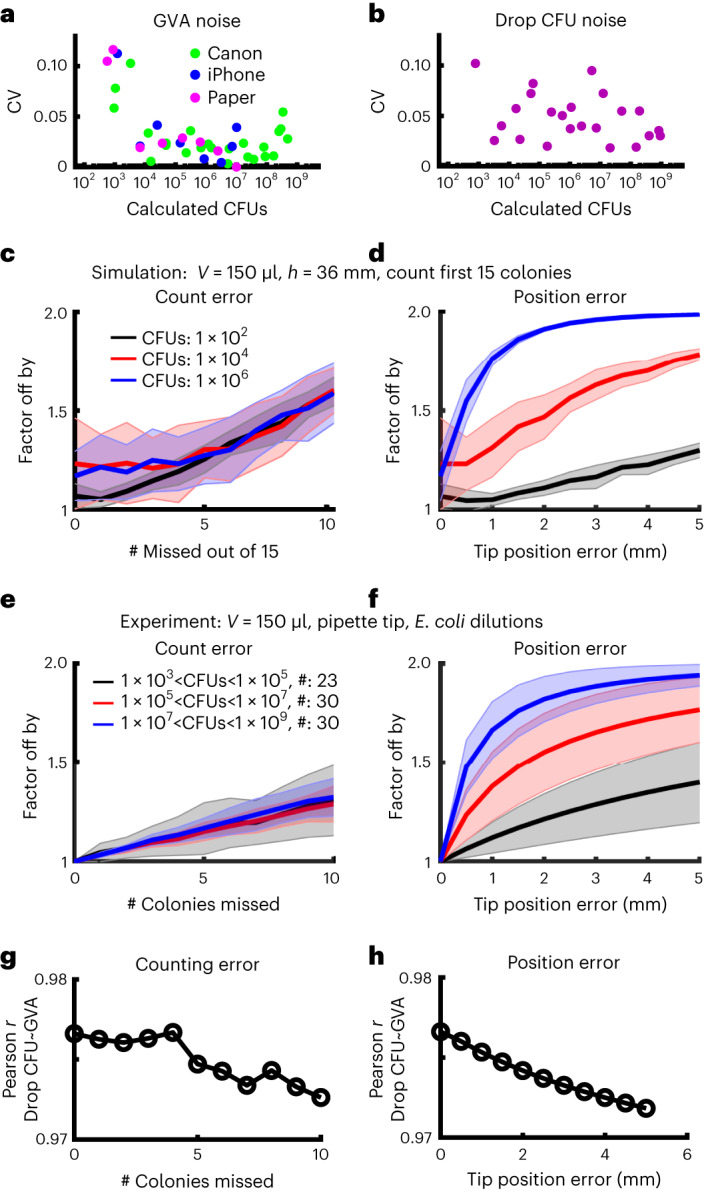
GVA has a low noise profile and is robust to missing colonies or tip position errors. **a**,**b**, CV among 4 technical replicates for different CFU concentrations for GVA using the Canon, iPhone or paper optical configuration (**a**) and the drop CFU (**b**). Technical replicates were used to quantify the noise intrinsic to the technique. **c**,**d**, The factor by which GVA calculation differs from the correct value as a function of the number of missed colonies (**c**) or error in tip position (**d**) in simulated results (see Methods). Solid lines and shaded error bars depict the mean ± s.d. of 1,000 simulations. **e**,**f**, Same error calculations for experimental data. Solid lines and shaded error bars depict the mean ± s.d. of all the pipette tips (#) included in each bin. **g**,**h**, Correlation between the GVA and the drop CFU assay as a function of counting and position errors.

To profile the technical noise inherent in GVA, we measured the count noise among 4 technical replicates across CFU concentrations ranging between 10^2^ and 10^7^ CFUs ml^−1^ (Fig. 4a,b). Noise was calculated using the coefficient of variation (CV) among replicates. Across all measured CFU concentrations, GVA noise is less than or equal to the noise of the drop CFU assay for the Canon, iPhone and paper optical configurations. As with the drop CFU assay, the GVA noise is heteroscedastic, increasing as the number of colonies decreases as expected for a Poisson process.

**Extended Data Fig. 8:**
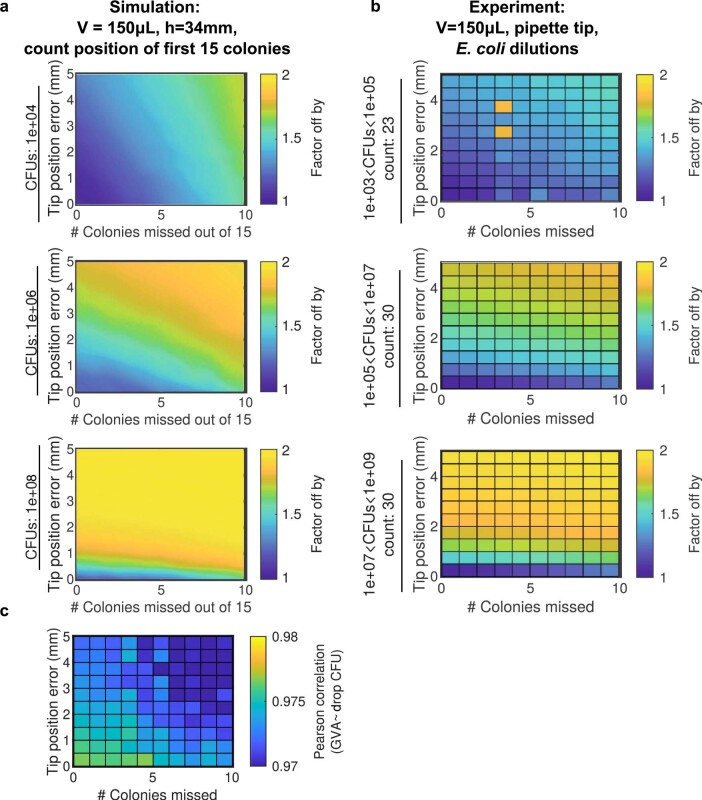
Sensitivity analysis of GVA calculations to error in missing colonies and location of the tip. **a)** Heatmap of the error as a function of both tip position and missing colony errors. **b)** Same analysis as in panel a, but with experimental data. CFUs/mL binned between 1e3 and 1e5 (top row), 1e5 and 1e7 (middle row), and 1e7 to 1e9 (bottom row). The number of pipette tips included in each bin is annotated by the count. **c)** Heatmap of the Pearson correlation between the drop CFU and GVA for both tip position and missing colony errors.

After confirming GVA’s low technical noise, we investigated the impacts of two types of real-world errors on GVA calculations: missing colonies and uncertainty in the position of the cone tip. These errors were examined using both simulated and experimental data. Predictably, as the number of missed colonies increases, the error increases (Fig. 4c,e) although the fractional error is the same in all seeding densities. Remarkably, eliminating 10 out of 15 counted colonies in the simulated data resulted in estimates within a factor of 2 of the correct answer, regardless of the initial CFU concentration. This robustness was recapitulated in the experimental data and is in agreement with the observation that the position of only 5 colonies is sufficient to calculate the CFUs ml^−1^ within a factor of 2 on average (Fig. 1e). For pipette tip position errors, the GVA calculations at high CFU concentrations are more sensitive to misidentification of the tip position than at low cell concentrations (Fig. 4d,f, blue versus black lines). Nevertheless, missing the tip position by 10% (4 mm for a 36 mm cone) still resulted in an estimate within a factor of 2 from the correct value in both simulations and experiments. Finally, the correlation between the drop CFU and the GVA (Fig. 1h) decreased from 0.98 to 0.97 for combinations of missing up to 10 colonies and missing the tip position by 4 mm (Fig. 4g,h and Extended Data Fig. 8).

In total, our analyses find that GVA is accurate and robust, retaining sensitivity over comparable ranges to the gold-standard drop CFU while reducing the cost and time.

### Proof of concept for high-throughput screen against stationary *E. coli*

Previous studies have found that slow growth is a non-inheritable form of antibiotic tolerance, buying time for viable cells to develop genetic resistance^20–24^. To explore the GVA technique’s potential for high-throughput viability measurements, we screened the ICCB Enzo Bioactive library (469 compounds) against stationary and exponentially growing cultures (Supplementary Figs. 1–3). Including controls and removing pipette errors, 2,267 viability measurements were accomplished. The equivalent screen using the drop CFU or spiral plater assays would have required 355 tip boxes or 2,267 Petri dishes, respectively. GVA required 24 tip boxes.

From the screen, we identified diphenyleneiodonium (DPI), a promiscuous NADPH oxidase (NOX) inhibitor^25^, to be active against both stationary and growing cultures; however, DPI is 10× more potent against exponentially growing cells—a difference in potency that was recapitulated in the drop CFU assay. Previous studies have identified DPI as possessing antimicrobial characteristics^26,27^; however, the mechanism of DPI bactericidal activity remains unknown. We were intrigued by DPI’s bactericidal activity as its reported activity in eukaryotes is the reduction of reactive oxygen species (ROS) by inhibiting NOXs^25^, which contrasts with the mechanism of many antibiotics which increase pools of reactive oxygen species (ROS)^28–30^. Subsequent investigations revealed that DPI increases intracellular superoxide in *E. coli*, as measured using CellROX dye^31^ (Supplementary Figs. 5 and 6). The intracellular ROS activated the SOS response pathway leading to filamentation^32,33^ (Supplementary Figs. 5 and 7). Using GVA to run a functional genomics screen, we found that recA-mediated activation of the SOS response was the key determinant of DPI bactericidal activity rather than the recently identified NOX-like genes in *E. coli*^34^ (Supplementary Figs. 5 and 8). Finally, viability, but not growth inhibition, checkerboards revealed that DPI exhibits strong antagonism against ciprofloxacin and gentamicin as observed for other redox-active prodrugs^35,36^ (Supplementary Figs. 5, 9 and 10).

## Discussion

One of the most surprising features of GVA was how well the theory enabled accurate viability estimates in practice regardless of the optical configuration. In simulations and experiments, errors in the colony count and tip position did not substantially alter CFU estimations when considering the experimental dynamic range. Furthermore, pipette tips are not perfect cones; small imperfections in manufacturing were visible at high magnifications. Despite these real-world variances—using an imperfect cone, selecting a few colonies and approximating the tip location—GVA still reproducibly and accurately calculated CFU concentrations across 6 orders of magnitude. This robustness emerges from utilizing the latent information encoded in a colony’s position.

Another unexpected feature of GVA was the observation of self-limiting colony size depending on the CFU density. As the concentration of colonies increased, the commensurate decrease in colony size preserved colony discreteness even for dense samples. Colony size, which varied in the strains tested, plateaued after overnight incubation and did not change over several additional days. The physiological basis of this phenomenon remains unknown, although we speculate that it could be due to a combination of nutrient limitation, quorum sensing and mechanically inhibited growth. However, the self-limiting colony growth in 3D may not be universally true for microbes, particularly for hyphae-forming fungi, which would limit the applicability of GVA. In addition, the decreasing colony size necessitated a high-resolution camera to quantify CFUs at the highest densities. These optical requirements limit the dynamic range, but not the accuracy, of field-deployable GVA protocols such as the smartphone or paper-based measurements. Another benefit of the density-dependent colony size is the reduced impact of contaminating microbes. In 2D, these contaminating species can grow unchecked, commonly resulting in complete loss of data, but in 3D they do not spread throughout the tip and can be identified by colony morphology.

GVA suffers from the same culturability limitations as the drop CFU^37,38^. In addition, it is unknown how many organisms that grow in 2D will not grow in 3D or vice versa. However, GVA worked for all commonly used laboratory strains tested as well as more complex samples such as biofilms and samples from human-associated biomes. Because GVA uses the same growth substrate as historic 2D culture techniques (for example, agarose and solid media), we anticipate many of the methods that have evolved to selectively culture different strains in Petri dishes will be transferable to GVA. Growth in 3D may also alter antibiotic sensitivity due to mechanosensitive changes in physiology^39,40^; therefore, more testing is required to compare the minimum inhibitory concentration values for 2D vs 3D plating. In addition, the size of the pipette tip and sample volume used in the assay dictate the limit of detection as well as the noise profile. However, the mathematics of GVA is applicable to p1000 or even larger tips maintaining a cone shape that could be readily quantified using a custom-generated paper ruler. Finally, the transient thermal shock of the current protocol using agarose did not impact the viability of the tested strains, but it could be a non-trivial perturbation for certain species. We found that low-melt agarose could be substituted in the GVA protocol for temperature-sensitive specimens at the cost of slightly longer gelling times in the pipette tip (6 s vs 12 s; see Methods). Another alternative could be the use of other hydrogels that crosslink via chemical reactions, such as sodium alginate.

In both the drop CFU and spiral plater methods, the incubation time remains a rate-limiting step, commonly taking at least overnight for visible colonies to emerge. For GVA, incubation is also a rate-limiting step; however, we achieved colony detection across all CFU concentrations within 8 h for *E. coli*. This improvement in time to detection is due to the unique optical configuration, the presence of a staining dye and the 3D geometry which maximizes light scattering. Decreasing the time further could be achieved with the use of fluorescent imaging. Although we expect time to detection for *E. coli* to be the experimental floor, this proof-of-concept data suggest that GVA could be a means to reduce the time of clinical antibiotic sensitivity profiling.

In total, we find the GVA approach to substantially reduce the time and reagents required for measuring cell viability compared with the established drop CFU assay while maintaining the same dynamic range, quantitative nature and versatility across different species that have made the drop CFU assay the gold standard for viability measurements in microbiology.

## Methods

### Protocols, software, hardware and example datasets

To facilitate the dissemination of GVA, we have constructed a website (https://www.colorado.edu/lab/chatterjeelab/geometric-viability-assay-gva) containing written and video protocols for sample embedding, imaging and GVA calculations, as well as associated software for all the methods described herein. For the Canon camera configuration (Extended Data Fig. 2), a complete set of build instructions, parts list and circuit design are included on the website. For the smartphone version, the CAD files for the 3D-printed parts are included, as well as instructions on printing configuration. For the paper-based version, a compiled MATLAB app (Windows only, no MATLAB license required) and the source code (all operating systems, MATLAB license required) for generating ruled grids for arbitrary cone geometries are included. The MATLAB software for segmenting the pipette tip images taken using either the mirrorless camera or the smartphone version is also available in either a compiled version (Windows only, no MATLAB license required) or the source code. This software can also be used to control the optical configuration using the Canon camera. Finally, example datasets from both the iPhone and Canon camera configurations are included for download.

### Strains and growth conditions

*E. coli* strain BW25113 (Yale Coli Genetic Stock Center) was used unless otherwise noted in the text. *E. coli* was grown in LB medium (Sigma Aldrich) at 37 °C in a shaking incubator. *B. subtilis* strain W168 was a gift from Ethan Garner and was grown in LB at 37 °C in a shaking incubator. *P. putida* strain KT2440 was a gift from Jacob Fenster and was grown in LB at 30 °C in a shaking incubator. *S. enterica* strain SL1344 was a gift from Corrie Detweiler and was grown in LB at 37 °C in a shaking incubator. *S. cerevisiae* strain BY4741 was a gift from Roy Parker and was grown in YEPD at 30 °C in a shaking incubator. *P. aeruginosa* strain PA01 was a gift from the Zemer Gitai and was grown in LB at 37 °C in a shaking incubator. Knockouts were selected from the Keio collection (Dharmacon). The PEC promoter library^41^ in *E. coli* was acquired from Dharmacon (PEC3877).

All bacterial and yeast strains were streaked onto an agar plate with appropriate antibiotic selection if required (kanamycin for Keio and PEC strains). These plates were kept for up to 1 month in a 4 °C refrigerator. Individual colonies were then selected and grown overnight in 3–5 ml LB with appropriate antibiotic selection if required. Each colony selected was considered a biological replicate. Multiple measurements of the same culture were considered technical replicates.

### Antibiotic treatments

Antibiotic treatments were typically performed in 96-well plates. For stationary-phase treatments, bacterial cells were grown overnight (≥16 h) in a shaking incubator (180 r.p.m.). For *P. putida* only, cells were grown for 2 d. Stationary-phase cells were distributed into 96-well flat-bottom plates with 100 µl of cells per well. Drug treatments at 1,000× were plated into a separate 96-well round-bottom plate. A 100 nl pin transfer was used to dilute the drug plate into the cell plate at a 1:1,000 ratio. This plate was then placed into a shaking incubator for the experimental time.

To measure antibiotic treatments in the exponential phase, overnight culture was diluted 1:1,000 into fresh LB. This culture was then placed into the incubator for 2 h. After this incubation, the cells were then distributed to the 96-well plate followed by drug treatment.

### Drop CFU assay

Drop CFU assays were performed as previously described^19^. Briefly, in a 96-well plate, 90 µl was added to all wells except row A. Into row A, a 100 µl volume of sample solution was added. From row A, 10 µl of cells were taken and added into row B, followed by 3 mixes. This process was repeated from B to C, until the final dilution on row H corresponding to a 1 × 10^−7^ dilution from the original sample. Pipette tips were changed for each row to reduce sample carry over between rows. From each well of the dilution series, 3 µl drops were transferred onto an LB–agar pad. Once all the liquid was absorbed into the agar (typically 15–30 min), the agar plates were inverted and placed into a 37 °C standing incubator overnight. The next morning, counting was performed manually. The first dilution with individually resolvable colonies was counted and multiplied by the corresponding dilution factor.

### Embedding for GVA

The goal of embedding was to have a uniformly mixed sample in a liquid hydrogel that would quickly solidify the 3D mould. We used 0.5% agarose as a convenient hydrogel that would solidify quickly and prevent cell motility once solidified. Pipette tips (200 µl, VWR universal) were used here as a reproducible and cheap 3D geometry scaffold. A detailed experimental protocol is available in Supplementary Information as well as on the GVA website (https://www.colorado.edu/lab/chatterjeelab/geometric-viability-assay-gva). Briefly, the protocol comprised 4 steps:

1. Preparing the agarose solution. A 0.66% agarose solution was prepared in the cell medium of choice. We found that the colour of LB, YEPD and blood (5% defibrinated sheep’s blood) did not substantially reduce colony visibility in the pipette tips. The agarose–media mixture was microwaved until completely dissolved. A careful watch was maintained during the heating to ensure the agarose did not boil over. Once the agar was fully dissolved, the liquid was placed in a 50 °C heat bath to maintain its liquid state until ready to use. For low-melt agarose, the liquid was placed in a 37 °C incubator until ready to use. At this stage, TTC (25 µg ml^−1^ final concentration) was added to the LB–agarose mixture from a 1,000× stock for all bacteria experiments. Respiring bacteria reduce tetrazolium to water-insoluble formazan, staining the colonies red.
2. Preparing the cells. A fresh 96-well round-bottom plate was prepared by adding 50 µl of media to each well. A pin transfer tool (hanging drop, VP409) was used to transfer 2 µl of the treated plate into the 50 µl media plate. If conducting a time-course experiment, the sample plate was then placed back into the shaking incubator.
3. Embedding. To embed, we found that a 12-multichannel pipette was the most convenient for high numbers of samples. We found a manual pipette easier to use, allowing for more control and reduced bubble formation during the mixing steps, but an electronic 12-channel pipette was also used successfully. The following items were gathered before pouring the liquid agarose into a reservoir: the 96-well plate with 50 µl samples (from step 2), a box of autoclaved P200 pipette tips, an empty P200 tip box filled with ice water, an empty P200 tip box. At this point, the liquid agarose was poured into a 100 ml reservoir for easy use with the multichannel pipette. Of the LB–agarose solution, 150 µl was taken from the reservoir and mixed twice with 1 row of the sample plate (200 µl final volume, 0.5% final agarose concentration, 1:100 final dilution of sample). After mixing, 150 µl was aspirated into the same pipette tips avoiding bubble formation. These tips were then placed into the ice bath for 6 s to ensure the hydrogel was solidified to plug the tip. For low-melt agarose, we increased the ice bath time to 12 s. Then the tips were ejected into the empty pipette tip box. This process was repeated for all 7 additional rows in the plate. Using the 150 µl volume and the 1:100 dilution from the original sample gave a lower limit of detection of 667 CFUs ml^−1^.
4. Incubation. Upon completion of the embedding process, the tip box with the LB–agarose–cell suspension was left at room temperature for ~5 min to ensure that the agarose was fully solidified. The tips were then moved into a standing incubator overnight for the colonies to grow. We found that the colonies did not continue to grow after overnight incubation, so the pipette could be imaged up to 4 d post embedding as long as they were maintained in a humid environment.

### Comparing cost and time of viability assays

#### Preparation time

The time to prepare the growth media (Fig. 3b) for the spiral plater and drop CFU includes: (1) autoclaving the agar, (2) cooling post autoclave, (3) plate pouring and (4) plate cooling. In GVA, agarose was melted in a microwave and subsequently equilibrated in a warm bath for 1 h before use.

#### Sample plating time

The sample plating time (Fig. 3c) for the spiral plater assay was based on the industry-reported value. Drop CFU and GVA were timed by an expert user using a 12-channel pipette and changing tips at every dilution and plating step.

#### Imaging and counting time

The time for quantification of samples (Fig. 3d) for GVA includes imaging (7 min for Canon with motorized stage and 30 min for iPhone), image processing and tip segmentation (5 min), and semi-automated colony counting (10 min) for 96 pipette tips. Spiral plater time was based on the industry-reported value using an automated colony counter. The drop CFU and paper GVA colonies were counted and recorded manually.

#### Agarose requirements

Twenty-five ml of 1.5% agar per 15 cm Petri dish was assumed for the drop CFU and spiral plater assays. GVA used 200 µl of 0.5% agarose per sample.

#### Instrument costs

The instrumentation costs for the spiral platers were based on quotes for both the plating instrument and automated imaging system from 3 manufacturers (see Supplementary Table 1). GVA instrument costs included the Canon camera, the 100 mm f/2.8 macro lens, and all the electronics and hardware to assemble the optical system. An electronic multichannel pipette was assumed for the drop CFU.

### Drug screen

A screen was performed with the ICCB Enzo Bioactive hits library (Enzo, BML-2840-0100). An overnight culture of 60 ml LB was grown to stationary phase with *E. coli*. The next morning, 60 µl of the overnight culture (stationary phase) was added to a fresh 60 ml of LB and grown for 2 h in the shaking incubator (exponential phase). The cells were then dispensed in 100 µl volumes into 96-well plates.

For comparing the dose responses for mitomycin C and DPI between the GVA and drop CFU (Supplementary Fig. 1), we fit the 4-parameter Hill equation to log-transformed viability data:

$$E={E}_{\max }+\frac{{E}_{0}-{E}_{\max }}{1+{\left(\frac{\left[{\rm{drug}}\right]}{\rm{E{C}}_{50}}\right)}^{h}}$$

using a bounded nonlinear least-squares regression function (‘curve_fit’) encoded in the ‘scipy’ Python package. Bounds for the no drug effect (*E*_0_), maximal drug effect (*E*_max_), potency (EC_50_) and Hill slope (*h*) were [8,10], [2.5,3], [0,10] [0.1,10], respectively. Complete parameter fits for all conditions are included in Supplementary Table 2.

### Biofilm growth and treatment

MG1655 *E. coli* strains were used for biofilms. Overnight cultures were diluted 1:10^5^ in LB. Biofilms were seeded in a U-bottom 96-well plate and grown for 48 h at 37 °C in a stationary incubator. For temporal experiments, a separate plate was used for each timepoint and biofilms were dispersed at the indicated times. Reported time represents the number of hours after the initial 48 h incubation. To disperse the biofilms, non-adhered cells were aspirated, wells were washed with PBS and fresh PBS was added to the wells. The plate was covered with foil plate seals (VWR, 60941-126) and put on a plate shaker at 3,000 r.p.m. for 30 min. Dispersed cells were diluted 1:10,000 and GVA was performed. A crystal violet stain was used to confirm proper dispersal; any replicates that were not fully dispersed were discarded.

### Imaging GVA tips

Imaging took place on a custom instrument (Extended Data Fig. 2) or an iPhone 12 (Fig. 2 and Extended Data Fig. 7). Parts list, circuit design and fabrication instructions for the GVA optical system are available on the GVA website (https://www.colorado.edu/lab/chatterjeelab/geometric-viability-assay-gva). Briefly, for the custom instrument, a mirrorless commercial camera (Canon EOS RP) with a 1:1 macro lens (Canon, f/2.8 100 mm) was used to obtain high-quality images that could resolve the smallest colonies. With this setup, we computed a pixel size of 6.6 µm (Extended Data Fig. 2c). To place the tips into the lightbox, a 12-channel P200 pipette was used. The camera with a macro lens was mounted on a V-channel linear rail opposite a linear stepper motor (NEMA 23). The stepper motor was controlled using a microcontroller board (Arduino Uno).

On the stepper motor, we mounted a custom 3D-printed lightbox that held the LEDs and optical diffusion plate for illuminating the pipette tips in *trans*. The 3D-printed parts were printed with polylactic acid using fused deposition modeling on a 3D printer (Lulzbot Pro). The print bed temperature was set to 70 °C for all layers and the nozzle temperature was set to 225 °C. Print speed was set to 10 mm s^−1^ for initial layers and then increased to 30 mm s^−1^ for subsequent layers.

Image acquisition was controlled using the MATLAB software which interfaced with digiCamControl (www.digicamcontrol.com) to access camera functions and acquire images. Typical camera settings used a shutter speed of 1/1,000 s, aperture of 2.8 and ISO of 100. The images were stored directly on the instrument computer as high-resolution.jpg files. Using this instrument, a typical experiment of 96 tips could be imaged in ~7 min.

For the iPhone, the 3D model files are available from the GVA website. Printing was done using the same configuration as for the LED lightbox. Post printing, the depth channel (green in Extended Data Fig. 7) was tapped with an 8–32 bit. After the holder was assembled on the Xenvo macro lens with the wide-field lens removed, the tip was positioned in front of a white backdrop and imaged with ambient illumination using the iPhone’s autofocus function. Three images per tip were taken and the tip most in focus was selected before processing using the MATLAB app.

### Image processing

The goal of the image processing was to extract individual pipette tips from the collected images and identify individual colonies. These were broken into two steps performed sequentially. MATLAB (Mathworks, R2022a) was used for all image processing analyses. The app can be used without a MATLAB license using a compiled version (Windows only) or on all OS using the source code.

1. Pipette tip segmentation. All images from a given field of view were converted to a 16-bit greyscale image. The overall orientation of the image was calculated to ensure that each tip was oriented perpendicular to the *x* axis. Due to small variations in the tip loading onto the lightbox, this was necessary to accurately calculate the colony distance from the pipette tip. The Hessian (fibermetric.m) of the image was calculated and convoluted with a horizontal line to locate the angle of the tips. The image was then rotated (imrotate.m) by this angle to orient the pipettes vertically in the image. To identify the *x* pixels corresponding to the pipette tip, the Hessian was again calculated from the rotated image. From the middle of the image, a convolution of a single line at different angles was used to calculate the left and right boundaries of the pipette tip. These lines were then extended to the bottom of the pipette tip to locate the left and right boundaries of the tip.
2. Semi-automated colony segmentation. Colonies were segmented using a semi-automated custom script in MATLAB. From the extracted image of the pipette tip, the user selected 1 of 5 different segmentation routines corresponding to the varying sizes of colonies in the pipette tip. The first routine segmented the entire pipette tip, while the last segmentation algorithm zoomed into 1/20th of the full tip and segmented the first 30 colonies. Segmentation was done using MATLAB’s Image Processing Toolbox. Subsequently, the user could curate the automated segmentation by adding missed colonies or removing erroneous colonies.
3. The colony count and position of the first and last colonies were used in equations (1) and (2) to calculate the GVA estimate of the CFU.s ml^−1^. For the error analysis, the factor by which the GVA estimate differed from the correct value was calculated according to: Factor off by = $\left(\frac{\left|{\rm{calculated}}-{\rm{actual}}\right|}{{\rm{actual}}}+1\right)$. This approach to error calculation takes into account the large dynamic range of possible CFUs ml^−1^.

### Microscopy measurements

For all microscopy experiments, cells from overnight cultures were diluted 1:100 in minimal media (PMM) and shaken for 2 h at 37 °C to ensure cells had exited the lag phase. After 2 h of growth, 2 µl of dilute cell culture was added to the top of a cooled, 200 µl 2% low-melt agarose pad with CellROX dye (5 µM). The agarose pad was moulded to fit in 96-well square-bottom plates (Brooks Automation, MGB096-1-2-LG-L). After 10 min of drying, the pad with affixed cells was inverted and pressed into the bottom of an imaging plate. Fields of view (FOVs) were selected manually on the microscope. After FOVs were selected and before the imaging started, the drug was added on top as done previously^19,42^. We have previously found that the drug diffuses through the pad within minutes.

Imaging took place using a Nikon Ti2 inverted microscope running the Nikon Elements software package. Fluorescent excitation was achieved with a laser source (488 nm and 561 nm) using a high-angle illumination to minimize the out-of-focus background. All images were acquired with a ×40, NA 0.95 air objective. Images were acquired on an sCMOS camera (Hamamatsu, ORCA-Fusion).

Image processing was done in MATLAB (Mathworks, R2020a) and followed the general scheme described in ref. ^19^. Briefly, the illumination profile for all images was estimated from the average of 50 images per FOV. Morphological opening and blurring were used to broaden the illumination pattern before correcting the images. After illumination correction, the jitter in the movie was removed by aligning each sequential frame using a fast 2D Fourier transform implemented in MATLAB. The background was locally subtracted on the basis of an estimation of the background computed using morphological image opening before segmentation.

Segmentation of cells was done using the Hessian-based ‘fibermetric’ routine implemented in MATLAB, which is specific for identifying tubular structures. Segmented regions were included only if they met minimum area and intensity thresholds, which were manually selected on the basis of the camera and laser settings. To remove rare, segmented debris, the mean Euclidean distance of each cell from all other cells in a multidimensional feature space was calculated and objects that were in the 95th percentile or above in average distance were removed^42^. A cell’s position in the feature space was defined by its segmented area, perimeter, major/minor axis lengths and circularity, extracted using MATLAB’s ‘regionprops’ command.

### Reporting summary

Further information on research design is available in the Nature Portfolio Reporting Summary linked to this article.

### Supplementary information

Supplementary Information Supplementary Tables 1–3 and associated legends, Movies 1 and 2 legends, Figs. 1–10 and associated legends, and derivation of PDF for a cone.

Reporting Summary

Supplementary Movie 1 Protocol for GVA sample embedding.

Supplementary Movie 2 Live cell imaging of CellROX stained cells imaged under agarose pad with different concentrations of DPI. Pad is made with PMM. Time (hh:mm) annotated in the upper left. DPI was added on top of the pad at the start of the video.

Supplementary Protocol 1 Detailed protocol for the GVA assay.

## Data Availability

The Enzo bioactive screen viability data are published on Zenodo at 10.5281/zenodo.7986816. All data needed to evaluate the conclusions in the paper are included in the paper and/or its Supplementary Information.

## Code availability

The MATLAB software used to segment the pipette tips and run GVA calculations is available from the GVA website (https://www.colorado.edu/lab/chatterjeelab/geometric-viability-assay-gva) alongside example datasets. The code is also available from Zenodo at 10.5281/zenodo.8342460. The use of the software is governed by the associated license.txt file in the code download.
