# Supplementary material for: A high-throughput and low-waste viability assay for microbes
Source: Nat Microbiol. 2023 Nov 2;8(12):2304–14. doi: 10.1038/s41564-023-01513-9 (PMC10686820; doi:10.1038/s41564-023-01513-9)
Supplement: Supplementary file 1 — Supplementary Tables 1–3 and associated legends, Movies 1 and 2 legends, Figs. 1–10 and associated legends, and derivation of PDF for a cone. [file 41564_2023_1513_MOESM1_ESM.pdf]

nature microbiology

Article https://doi.org/10.1038/s41564-023-01513-9

A high-throughput and low-waste viability assay for microbes

In the format provided by the authors and unedited

A High Throughput and Low Waste Viability Assay for Microbes

Christian T. Meyer[1,2] *, Grace K. Lynch[1], Dana F. Stamo[2], Eugene J. Miller[1], Anushree Chatterjee[2,3,4], and Joel M. Kralj[1] *

[1]BioFrontiers and MCDB Department, University of Colorado Boulder, Boulder, CO, USA
[2]Chemical and Biological Engineering, University of Colorado Boulder, Boulder, CO, USA
[3]Antimicrobial Regeneration Consortium (ARC) Labs, Louisville, CO, USA
[4]Sachi Bioworks, Louisville, CO, USA
*Correspondence should be directed to C.T.M (chme4702@colorado.edu) and J.M.K. (joel.kralj@colorado.edu).

Supplemental Materials

Contents

1 Supplemental Movies Legend **1**

2 Supplemental Tables **1**

3 Proof of Concept HTS Viability Screen **3**

4 Derivation of the axial probability density function for a cone. **16**

1 Supplemental Movies Legend

Supplemental Movie 1: Protocol for GVA sample embedding.

Supplemental Movie 2: Live cell imaging of CellROX stained cells imaged under agarose pad with different concentrations of DPI. Pad is made with PMM. Time (HH:MM) annotated in the upper left. DPI was added on top of pad at start of video.

2 Supplemental Tables

Table S1: Pricing for viability measurement consumables and Spiral Plater instrumentation.

**Consumables**				
Reagent	Source	Amount	Cost	Cat. Number
Biotix uTIP™, 200uL Low Retention	VWR	960 tips/case	$108.62	89425-644
BD Difco™ Plate Count Agar	Fischer Scientific	500 g	$190.60	DF0479-17-3
Disposable 15cm Petri dishes	VWR	500/case	$362.00	25384-092
96-well Cell Culture Plates	VWR	100/case	$597.80	10062-900
**Instruments**				
Instrument Name	Type	Cost		
easySpiral	Spiral Plater	$15,184.00		
Scan 300	Imager	$6,555.00		
Wasp Touch	Spiral Plater	$30,950.00		
ProtoCOL 3 Plus	Imager	$31,140.00		
Eddy Jet 2W	Spiral Plater	$26,000.00		
SphereFlash	Imager	$11,000.00		

Table S2: Fit parameters and quality for GVA and drop CFU-based dose-response curves for Mitomycin C and DPI against stationary phase and exponentially growing E. coli cells. See Methods for a description of the fitting algorithm.

Hill eq. Parameter	Drug	Growth Phase	Viability Method	Fit Value
No drug effect, E0 [CFUs/mL]	DPI	e^x	Drop CFU	9.36
			GVA	9.20
		stationary	Drop CFU	8.93
			GVA	9.11
	Mitomicin C	e^x	Drop CFU	9.10
			GVA	8.91
		stationary	Drop CFU	8.45
			GVA	9.06
Potency, EC50 [ug/mL]	DPI	e^x	Drop CFU	2.03
			GVA	1.08
		stationary	Drop CFU	19.53
			GVA	20.93
	Mitomicin C	e^x	Drop CFU	1.86
			GVA	2.72
		stationary	Drop CFU	5.65
			GVA	5.36
Efficacy, Emax [CFUs/mL]	DPI	e^x	Drop CFU	3.81
			GVA	5.41
		stationary	Drop CFU	2.52
			GVA	2.52
	Mitomicin C	e^x	Drop CFU	2.52
			GVA	2.52
		stationary	Drop CFU	2.52
			GVA	3.00
Hill slope, h	DPI	e^x	Drop CFU	1.33
			GVA	1.86
		stationary	Drop CFU	2.18
			GVA	0.96
	Mitomicin C	e^x	Drop CFU	3.46
			GVA	2.67
		stationary	Drop CFU	3.76
			GVA	1.45
Fit Quality, R^2	DPI	e^x	Drop CFU	0.91
			GVA	0.88
		stationary	Drop CFU	0.98
			GVA	0.94
	Mitomicin C	e^x	Drop CFU	0.97
			GVA	0.94
		stationary	Drop CFU	0.96
			GVA	0.92

Table S3: Number of GVA measurements for each drug class as a function of growth phase.

Culture	Drug class	Sample Count
Stationary	Bioactive lipids	210
	CNS receptor ligands	55
	DMSO	211
	Inhibitors	381
	Ion channel ligands	154
	Lipid biosynthesis	47
	Misc.	20
	Nuclear receptor ligands	46
e^x	Bioactive lipids	212
	CNS receptor ligands	56
	DMSO	213
	Inhibitors	383
	Ion channel ligands	154
	Lipid biosynthesis	46
	Misc.	20
	Nuclear receptor ligands	47

3 Proof of Concept HTS Viability Screen

Previous studies have found slow growth is a non-inheritable form of antibiotic tolerance buying time for viable cells to develop genetic resistance [1]. Slow-growing cells commonly have reduced metabolic activity [2] and DNA replication [3] as compared to exponentially growing cells. As a result, slow-growing cells are refractory to antibiotics targeting DNA synthesis (fluoroquinolones) [4], protein translation (aminoglycosides) [5], and cell wall biogenesis (beta-lactams) [6]. Growth-dependent tolerance can only be observed by measuring viability but the tedium and cost of the drop CFU assay limits extensive profiling. Using GVA, we directly compared the viability of exponentially growing cells and stationary phase cells to different doses of three antibiotics for varying amounts of time. In total, we tested 3 antibiotics at 6 different concentrations for 5 different durations against stationary and exponential cells, in duplicate, for a total of 360 viability measurements (Fig. S1a,b). This data was acquired by a single researcher in one day using only 4 tip boxes. Stationary phase cells were more resistant to ciprofloxacin, carbenicillin, and gentamicin. Particularly, for carbenicillin, there was less than a 10-fold decline in viability of stationary cells treated with 100 μg/mL carbenicillin for 24 hours, as compared to a 10,000 fold decrease in exponential cells. Treating exponential cells with 10 μg/mL carbenicillin showed no change in the number of colonies during the first 6 hours, followed by an increase in viable cells after 24 hours treatment indicative of a slowly-expanding, drug-tolerant pool (Fig. S1b). Ciprofloxacin at 10 μg/mL had a biphasic pharmacodynamic profile with initial bactericidal activity within an hour resulting in a 10-fold reduction in viability for both stationary and exponentially growing cultures. However, this activity stabilized through 6 hours and a second phase of killing was achieved by 24 hours. Gentamicin at 10 μg/mL required a full 24 hours to achieve more than a 10-fold reduction in stationary phase cell viability. For untreated cultures, we observed the concentration of exponentially growing cells increased till a peak concentration of $\sim 10^9$ CFUs/mL at 6 hours (Fig. S2). Once in stationary phase, the number of viable cells declined over time as previously reported [7]. These data exemplified the utility of GVA for measuring the efficacy of treatments agnostic to growth rate.

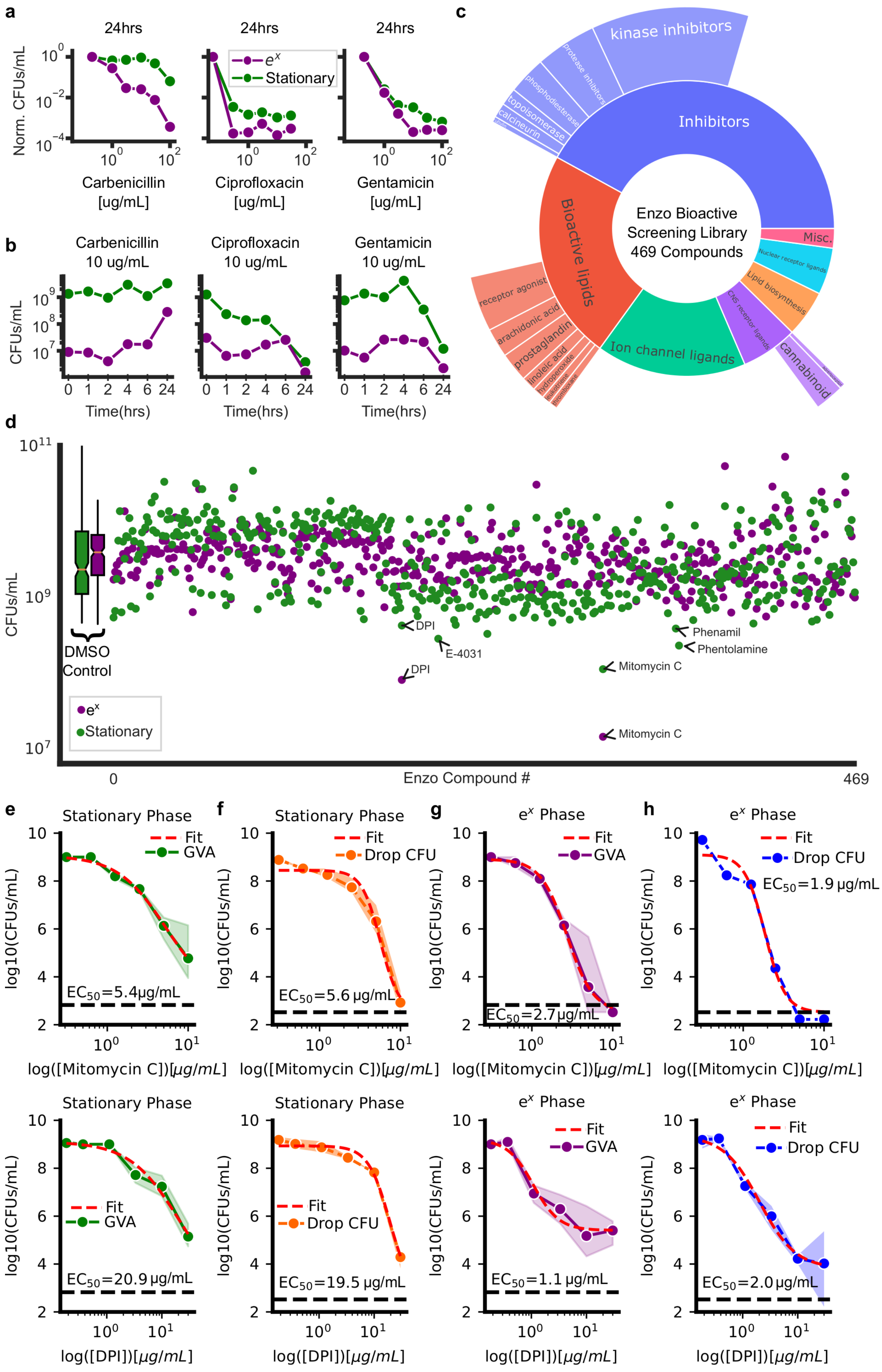
a
24hrs
Norm. CFUs/mL
Carbenicillin [ug/mL]
Ciprofloxacin [ug/mL]
Gentamicin [ug/mL]
Stationary
b
Carbenicillin 10 ug/mL
Ciprofloxacin 10 ug/mL
Gentamicin 10 ug/mL
CFUs/mL
Time(hrs)
c
Enzo Bioactive Screening Library 469 Compounds
Inhibitors
kinase inhibitors
protease inhibitors
phosphodiesterase
topoisomerase
calcineurin
Misc.
Nuclear receptor ligands
Lipid biosynthesis
CNS receptor ligands
cannabinoid
Ion channel ligands
Bioactive lipids
receptor agonist
arachidonic acid
prostaglandin
linoleic acid
hydroperoxide
leukotriene
thromboxane
d
CFUs/mL
DMSO Control
DPI
E-4031
Mitomycin C
Phenamil
Phentolamine
Stationary
Enzo Compound #
0
469
e
Stationary Phase
Fit
GVA
EC50=5.4μg/mL
log10(CFUs/mL)
log([Mitomycin C])[μg/mL]
f
Drop CFU
EC50=5.6 μg/mL
g
EC50=2.7μg/mL
h
EC50=1.9 μg/mL
EC50=20.9 μg/mL
EC50=19.5 μg/mL
EC50=1.1 μg/mL
EC50=2.0 μg/mL
log([DPI])[μg/mL]

Figure S1: **GVA screening of the Enzo library identifies DPI as active against stationary phase *E. coli*.** a) Dose-response of 3 antibiotics for stationary and exponential (e^x) cultures after 24 hours of treatment. Each point is the mean of duplicate measurements. CFUs/mL were normalized to an untreated control. b) Viability over time for stationary and exponential cells at one concentration of antibiotic. c) Drug classes of the Enzo Bioactive Screening Library. The size of the donut wedge is proportional to drug class representation. Targets of each class and relative representation are depicted on the outer ring. d) Absolute viability of stationary (green) and exponentially growing (purple) cells after 24 hours of treatment with Enzo library. Each condition was run in duplicate and the mean was taken in log space. e,f) Dose-responses for stationary phase *E. coli* at different concentrations of Mitomycin C (top) and DPI (bottom) measured using GVA (e) or drop CFU (f). The potency (EC_{50}) is based on the fit to the 4-parameter Hill equation (red line, see Methods). Hill equation was fit for log-transformed data. The markers and error bars indicate the mean of and standard deviation between 3 biological replicates, respectively, calculated after log transforming the CFUs/mL. The limit of detection (LOD) for the GVA and drop CFU methods are annotated with a dotted horizontal line. g,h) Dose-responses for exponentially growing *E. coli* for GVA (g) and drop CFU (h).

To explore the GVA technique's potential for high throughput viability measurements, we screened the ICCB Enzo Bioactive library (469 compounds) against stationary and exponentially growing cultures (Fig. S1c,d). The Enzo library has a wide breadth of chemical matter including bioactive lipids, small molecule inhibitors, and ion channel ligands (Fig. S1c) and spans the structural diversity of larger libraries like the Maybridge HitFinder library of approximately 14,000 compounds (Fig. S3a). Viability of BW25113 *E. coli* treated with the Enzo library was measured in both exponential and stationary phase. Including controls and removing pipette errors, 2267 conditions were measured. The equivalent screen using the drop CFU or Spiral Plater assays would have required 355 tip boxes or 2267 petri dishes, respectively. GVA required 24 tip boxes. No edge effects were observed for either stationary or exponential plates (Mann-Whitney U test, p-val>0.05, Fig. S3b). Average differences among drug classes were modest (Fig. S3c, p-val>0.001 ANOVA, p-val corrected for multiple hypothesis testing) and none significantly different than the control (p-val>0.01, Pairwise Tukey Test). Five compounds were selected for follow-up verification (mitomycin C, phentolamine, E-4031, phenamil, and diphenyliodonium) corresponding to a $\sim$1% hit rate. Mitomycin C is a known antibiotic acting through DNA cross-linking. As expected, we found it to be 2X more potent against exponentially growing cells compared to cells in stationary phase according to both GVA and drop CFU measurements (Fig. S1e-h, top). Potency (EC50) was measured by fitting the dose-response curves to a 4-parameter Hill equation (See Methods). Parameter fits are reported in Supplemental Table 2. E-4031, phenamil, and phentolamine did not have any dose-dependent effect on viability (Fig. S4). Finally, we found diphenyleneiodonium (DPI), a promiscuous NADPH Oxidase (NOX) inhibitor [8], to be active against both stationary and growing cultures (Fig. S1h); however, DPI is 10X more potent against exponentially growing cells (see Supplemental Table 2 for complete parameter fits). This difference in potency was recapitulated in the drop CFU assay. Previous studies have identified DPI as possessing antimicrobial characteristics [9, 10]; however, the mechanism of DPI bactericidal activity remains unknown. We were intrigued by DPI's bacteriocidal activity as it reduces Reactive Oxygen Species (ROS) in eukaryotes by inhibiting NOXs [8] which is in contrast to the mechanism of many antibiotics which increase ROS pools [11–13].

In order to investigate the bactericidal mechanism of DPI, we first examined *E. coli* ROS levels upon treatment with DPI. ROS levels were determined with the fluorescent CellROX dye which measures cytoplasmic superoxide [14]. Single cell fluorescence was measured over time after treatment with a lethal DPI dose and compared to an untreated control (Fig. S5a). As expected, DPI substantially decreased ROS reaching the nadir around 75 minutes after the drug was added (Fig. S5a compare blue and yellow lines, Supplemental Movie 2). The depth and duration of the ROS reduction was proportional

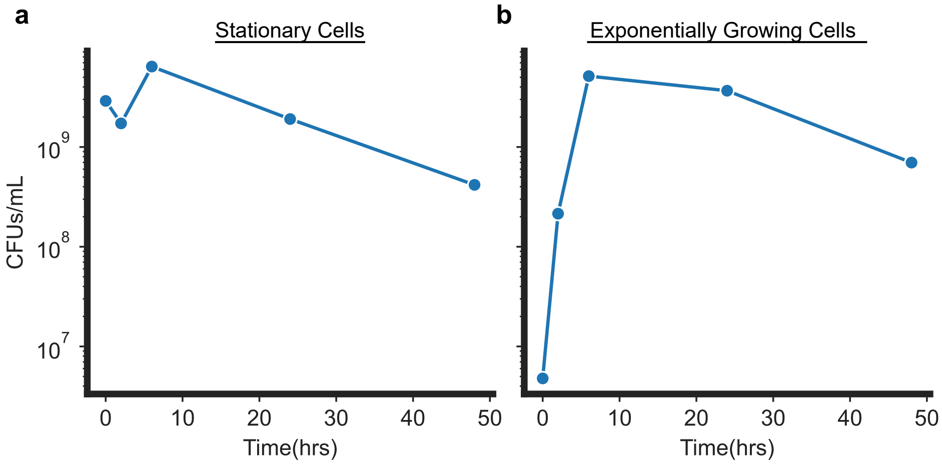

Figure S2: **Cell counts over time in stationary versus exponential cultures.** a) Number of CFUs/mL in stationary (a) versus exponential culture (b). To generate exponential culture, stationary phase cells were diluted 1:1000 in fresh LB media and place in the shaking incubator (180RPM) at 37 °C for 2 hours prior to beginning experiment.

to the DPI concentration (Fig. S6a). Surprisingly, this decrease was followed by a rapid spike in ROS. In contrast to DPI, ciprofloxacin treatment resulted in monotonically increasing levels of ROS (Fig. S5a, orange line). Increased levels of ROS underlie ciprofloxacin's bactericidal activity; therefore, we next investigated if the ROS spike induced by DPI also underlies its bactericidal activity. We compared DPI sensitivity of stationary phase cells in aerobic versus anaerobic environments. DPI was less active in anaerobic cultures (Fig. S5b), similar to gentamicin or ciprofloxacin (Fig. S6b,c). This data suggested high levels of ROS are part of the bactericidal mechanism of DPI, despite it initially decreasing ROS. In further support of this, adding a ROS scavenger also reduced DPI efficacy (Fig. S6d).

Intermediate DPI concentrations altered ROS levels but maintained viability when measured with GVA. We examined the cell morphology after 4 hours of treatment with less than 10 μg/mL DPI and observed the formation of bacterial filaments (Fig. S5c, Supplemental Movie 2). Filamentation is a classic hallmark of SOS activation [15] and increases in ROS are an established SOS activator [16]. Therefore, we wondered if DPI was activating SOS. We examined the promoter activity of genes downstream of lexA using the PEC GFP-promoter library [17]. LexA is a master transcriptional repressor of genes in the SOS regulon such as *polB*, *dinB*, *dinG*, and *yjiH*, and is auto-catalytically degraded by activated recA. We observed persistent, dose-dependent induction of the *polB* promoter compared to a ribosomal protein control (*rrnB*) (Fig. S5d, solid versus dashed lines). The highest promoter activity corresponded to an intermediate dose of DPI (3 μg/mL) where filamentation was observed. We also observed a DPI-dependent increase in *dinB*, *dinG*, and *yjiH* promoter activity (Fig. S7). The *lexA* promoter, which is self-repressed, also increased activity within 90 minutes of DPI addition.

Because SOS activity reduces the efficacy of other bactericidal agents [18], we predicted that recA-mediated SOS activation was critical for maintaining viability in the presence of DPI. As predicted, *recA* knockouts were more susceptible to DPI in both stationary and exponential phases of growth (Fig. S5e), though the increased DPI potency was more pronounced in exponentially growing cells. In contrast, knocking out other DNA repair enzymes, redox repair enzymes, or ROS scavengers did not substantially change the potency of DPI in either growth phase (Fig. S8). Knocking out *yedZ* and *fre*, genes recently identified as part of a NOX-like system in bacteria [19], modestly increased the potency of DPI against stationary cells indicating these proteins are unlikely to be the main target of DPI in *E. coli* (Fig. S5e). Therefore, our data showed DPI

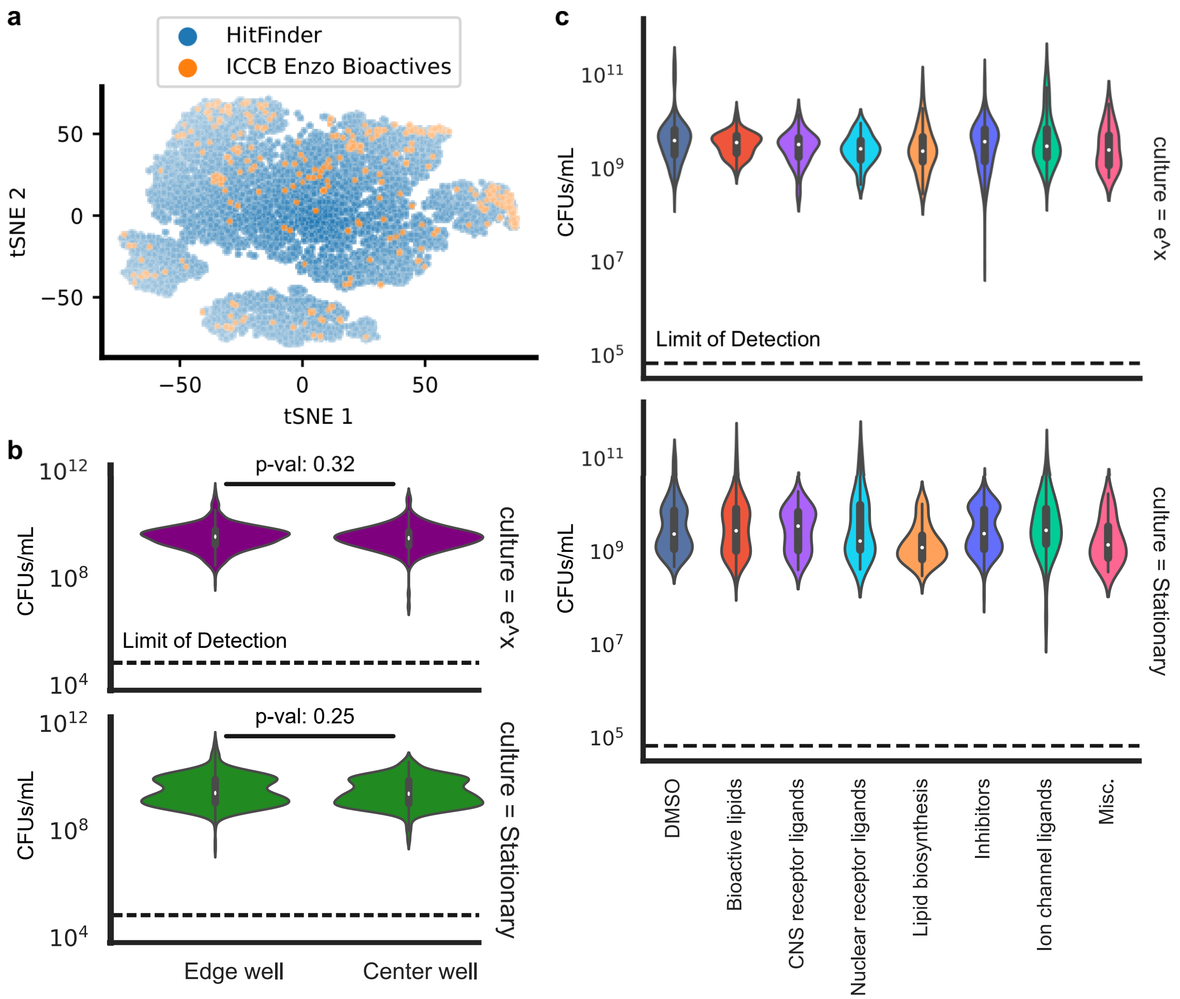

Figure S3: **Enzo screen controls.** a) Library diversity of ICCB Enzo Known Bioactive library compared to the Maybridge HitFinder library. Tanimoto similarity between all molecules based on SMILES was calculated using the RDKit package in python. From this distance matrix, the tSNE embedding was initialized with PCA and computed with a perplexity of 50. b) Distribution of CFUs/mL for conditions on the edge of the plate versus in the center wells for both stationary and exponential cultures. Statistical test used a Mann-Whitney U test for nonparametric distributions (p-val$>$ 0.05). The number of samples in the edge wells and center wells for the exponential culture was (228, 696) and stationary culture was (223, 696), respectively c) Distribution of CFUs/mL for different drug classes identified in the Enzo Library (See Fig. S1c). No class differences were found when using two-sided ANOVA (p-val$>$0.001, p-val corrected for multiple hypothesis testing). No differences from control were found using the Pairwise Tukey Test (p-val$>$0.01, Pairwise Tukey Test two-sided). The number of samples from each drug class are summarized in Table S3. In both b,c the boxplots show the mean (white dot), the inner quartiles (25%,75%, solid black box), outer quartiles (whiskers). No outliers were observed.

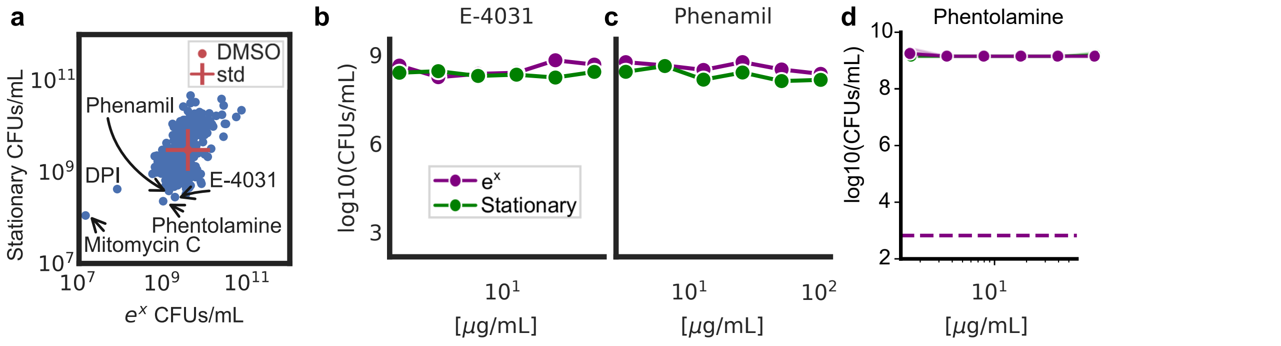

Figure S4: a) Scatter plot of stationary phase versus exponential phase from the screen. The standard deviation of DMSO controls are depicted with a red cross. Selected hits are annotated. b,c,d) E-4031 (b), phenamil (c), and phentolamine (d) dose-response curves against stationary or exponentially (e^x) growing cultures.

activated SOS, and that SOS activation enhanced cell viability.

We therefore wondered if DPI would antagonize other antibiotics whose efficacy is reduced by the SOS response. Such antagonism has been observed in combinations of ciprofloxacin with metronidazole, a redox-active prodrug known to activate SOS [20]. To test for antagonism, we measured viability in a time-resolved, checkerboard assay using GVA (Figs. S5f, S9). In the checkerboard assay, DPI was combined with either ciprofloxacin or gentamicin across a 6×6 dose matrix. The ease of GVA enabled sampling the checkerboard over time, resulting in a complete pharmacokinetic profile of the drug-drug interaction. DPI antagonized both ciprofloxacin and gentamicin against stationary phase *E. coli* increasing the viability 1,000-fold as compared to either drug alone after 24 hours treatment (Fig. S5f,g). This antagonism was not observed in a growth inhibition assay (Fig. S5h) emphasizing the value of viability data when investigating drug-drug interactions. DPI antagonism of ciprofloxacin and gentamicin was also observed in *S. typhimuirium* (Fig. S10). Cells pretreated with DPI for 2 hours before adding ciprofloxacin further increased protection, while pretreating with ciprofloxacin for 2 hours reduced DPI's antagonistic effects (Fig. S5i).

In total, we found DPI initially decreased ROS followed by a ROS burst which enhanced its bactericidal effects. As expected with previous studies of ROS lethality [16], the potency of DPI depended on SOS-activation mediated via recA. By activating SOS, DPI led to an increase in drug tolerance to fluoroquinolones and aminoglycosides as revealed by temporal viability checkerboards.

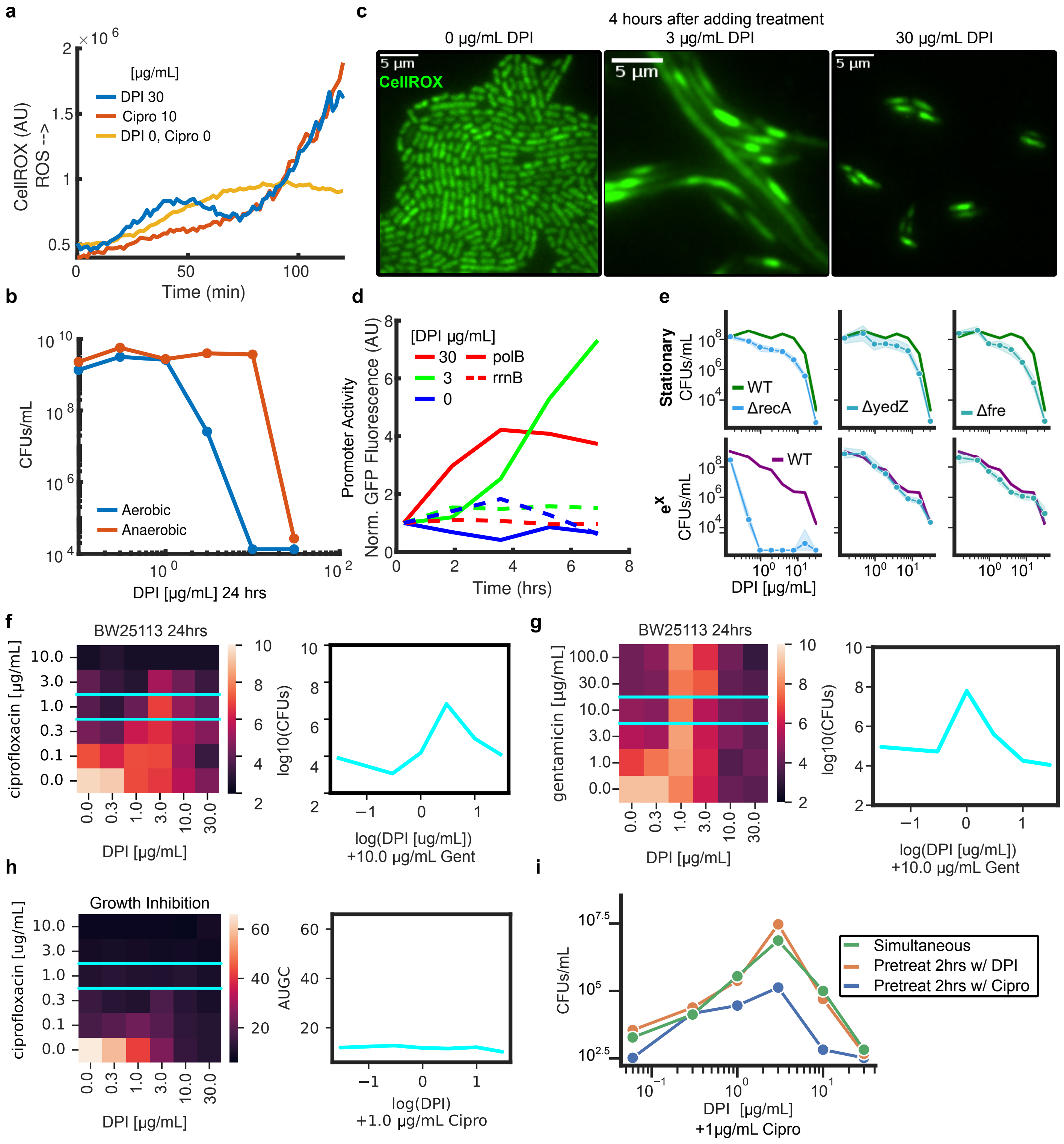
a
CellROX (AU)
ROS -->
[μg/mL]
DPI 30
Cipro 10
DPI 0, Cipro 0
Time (min)
c
4 hours after adding treatment
0 μg/mL DPI
3 μg/mL DPI
30 μg/mL DPI
5 μm
CellROX
b
CFUs/mL
Aerobic
Anaerobic
DPI [μg/mL] 24 hrs
d
Norm. GFP Fluorescence (AU)
Promoter Activity
[DPI μg/mL]
30
3
0
polB
rrnB
Time (hrs)
e
Stationary
CFUs/mL
WT
ΔrecA
ΔyedZ
Δfre
DPI [μg/mL]
f
BW25113 24hrs
ciprofloxacin [μg/mL]
DPI [μg/mL]
log10(CFUs)
log(DPI [ug/mL])
+10.0 μg/mL Gent
g
BW25113 24hrs
gentamicin [μg/mL]
DPI [μg/mL]
log10(CFUs)
log(DPI [ug/mL])
+10.0 μg/mL Gent
h
Growth Inhibition
ciprofloxacin [ug/mL]
DPI [μg/mL]
AUGC
log(DPI)
+1.0 μg/mL Cipro
i
CFUs/mL
Simultaneous
Pretreat 2hrs w/ DPI
Pretreat 2hrs w/ Cipro
DPI [μg/mL]
+1μg/mL Cipro

Figure S5: **DPI generates ROS, activates the SOS response, and antagonizes ciprofloxacin.** a) Median, single-cell CellROX signal as a function of time for DPI (blue), ciprofloxacin (orange), and an untreated control (yellow). b) Efficacy of DPI in aerobic and anaerobic conditions. See Fig. S6b,c for ciprofloxacin and gentamicin. c) Representative images of live *E. coli* cells stained with the CellROX dye for three DPI concentrations 4 hours after adding DPI. Similar results were observed in 3 independent experiments. Brightness and contrast is the same for all images. See Supplemental Movie 2. d) Measurement of *polB* and *rrnB* promoter activity normalized to t=0. e) DPI dose response for *E. coli* knockout mutants treated during stationary (top panels) or exponential growth (bottom panels). The dose response for the wild-type (WT) cells is depicted in green or purple, respectively. Shaded errorbars equal to the standard deviation in logspace between 3 replicates. See Fig. S8 for other mutants. f) GVA checkerboard assay for DPI combined with ciprofloxacin at 24 hours. Each square in the heatmap was the mean of duplicate conditions. Colorbar correspond to the log10(CFUs/mL) for each dose combination. Left panel shows the dose response for DPI plus 1 μg/mL ciprofloxacin (cyan). See Fig. S9 for full time series. g) GVA checkerboard assay for DPI combined with gentamicin at 24 hours. h) Growth inhibition checkerboard for DPI and ciprofloxacin. Optical density was measured for each condition over 8 hours and the integrated area under the growth curve (AUGC) is depicted (colorbar). i) Dose response curves for temporally staggered combinations. All treatments lasted for 24 hours total. Pretreated conditions were treated for 2 hours with a single drug followed by 22 hours with both drugs.

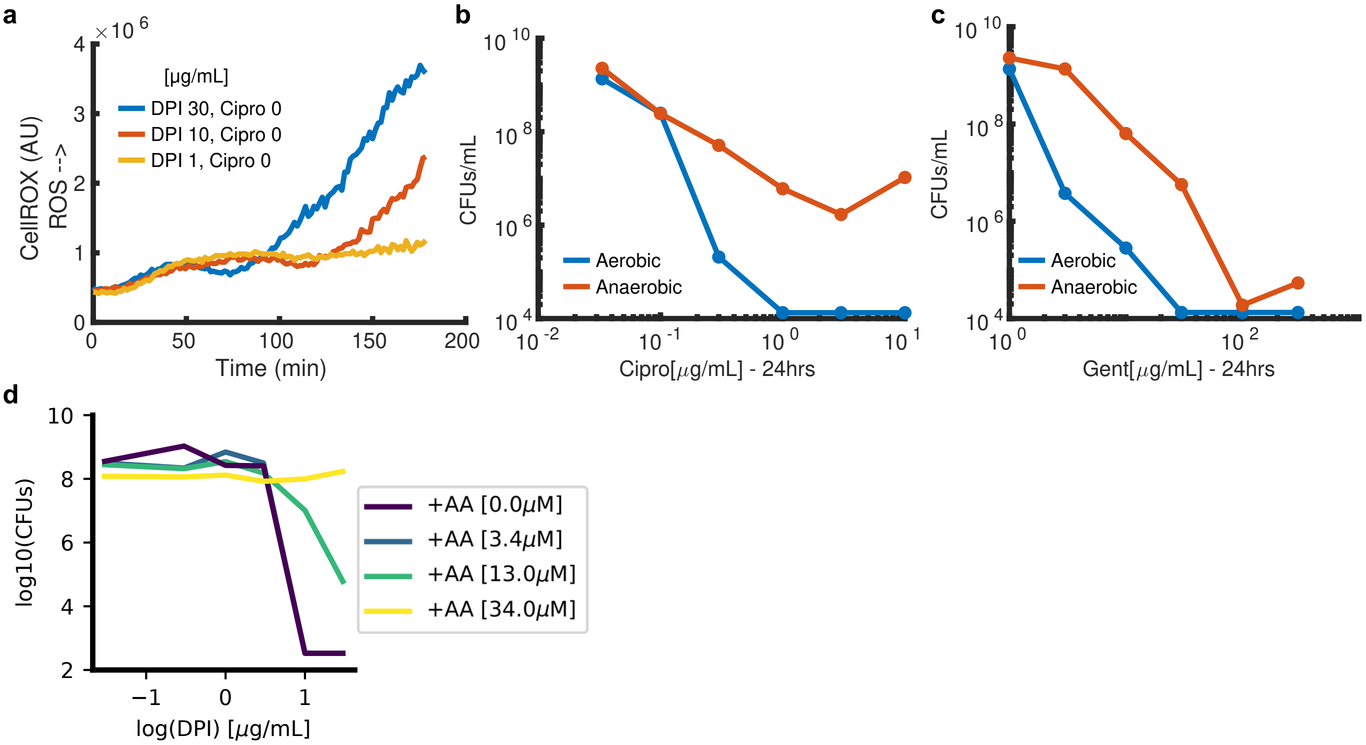

Figure S6: a) Duration of ROS reduction and onset of the secondary ROS spike is DPI-concentration dependent. Depicted is the median single-cell CellROX signal as a function of time for different concentrations of DPI. b,c) Dose response curve for ciprofloxacin (b) and gentamicin (c) against stationary phase cells in aerobic or anaerobic conditions. Treatment was for 24 hours. d) Efficacy of DPI as a function of increasing concentrations of the ROS-scavanger, ascorbic acid (AA).

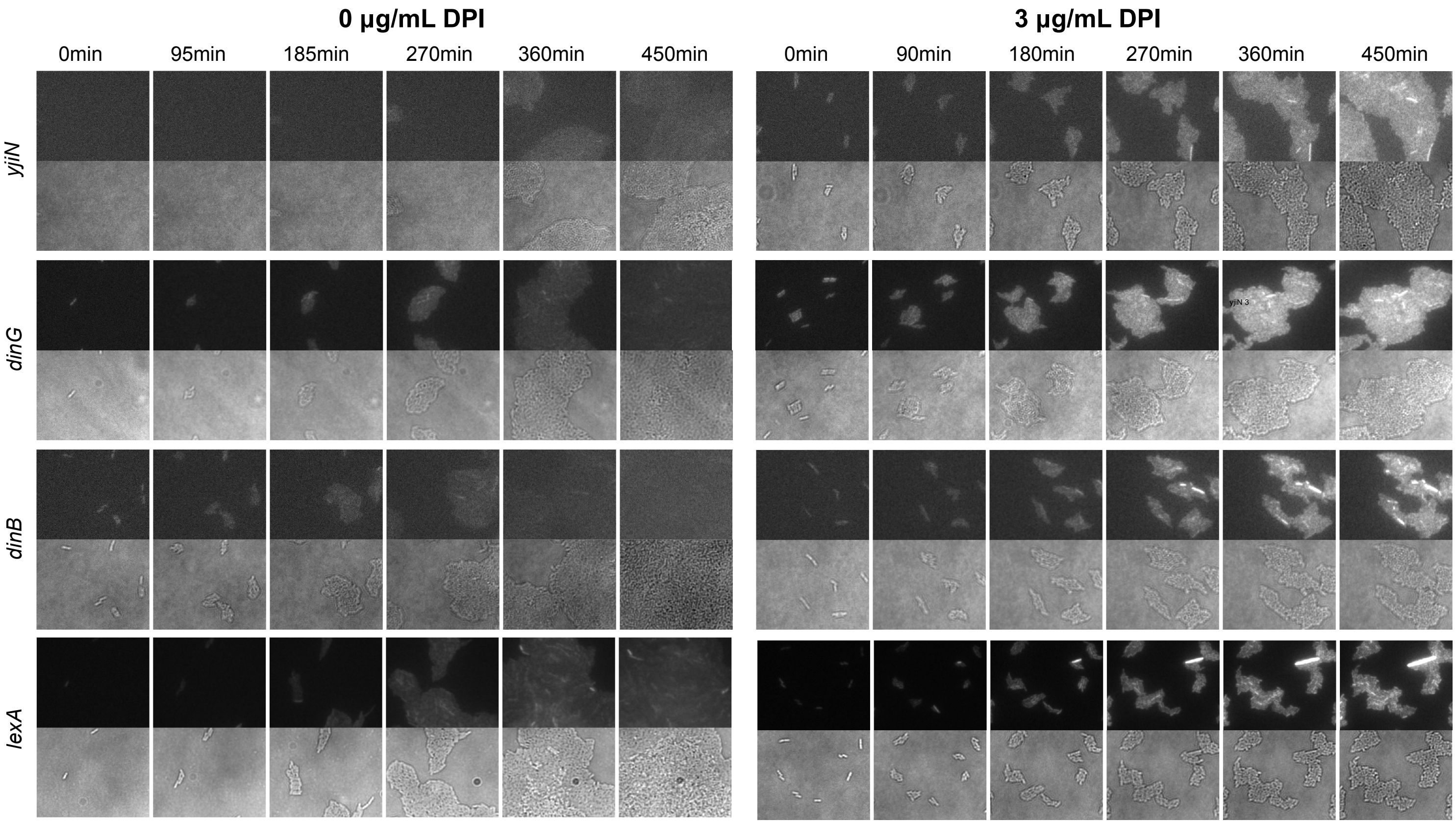

Figure S7: Strip charts of lexA-repressed genes (rows) using the PEC library. GFP fluorescence (top panels of each row) is proportional to each gene's promoter activity. Bottom panel of each row depicts brightfield image. Columns correspond to different timepoints post treatment.

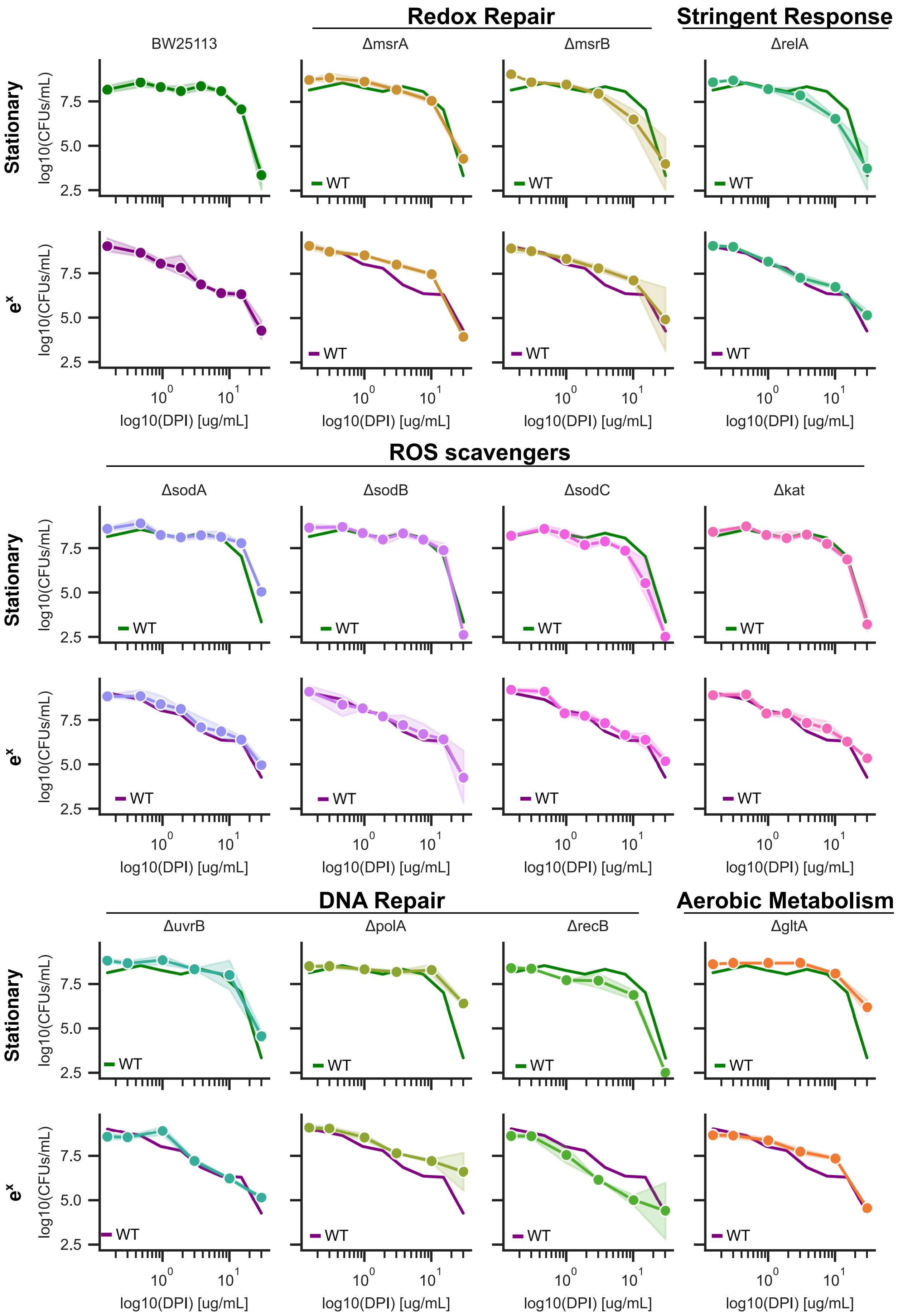

Redox Repair
Stringent Response
BW25113
ΔmsrA
ΔmsrB
ΔrelA
Stationary
e^x
log10(CFUs/mL)
7.5
5.0
2.5
WT
10^0
10^1
log10(DPI) [ug/mL]
ROS scavengers
ΔsodA
ΔsodB
ΔsodC
Δkat
DNA Repair
Aerobic Metabolism
ΔuvrB
ΔpolA
ΔrecB
ΔgltA

Figure S8: **Sensitivity of gene mutants to DPI in exponential and stationary phase.** Wild type reference depicted in solid line for each mutant. Error bars are the standard deviation in log space between 3 biological replicates with points denoting the mean. Mutants were selected from the Keio collection. Kanamycin (25 μg/mL) was included in the all Keio culture conditions both in the overnight culture and during treatment with DPI to maintain gene knockout.

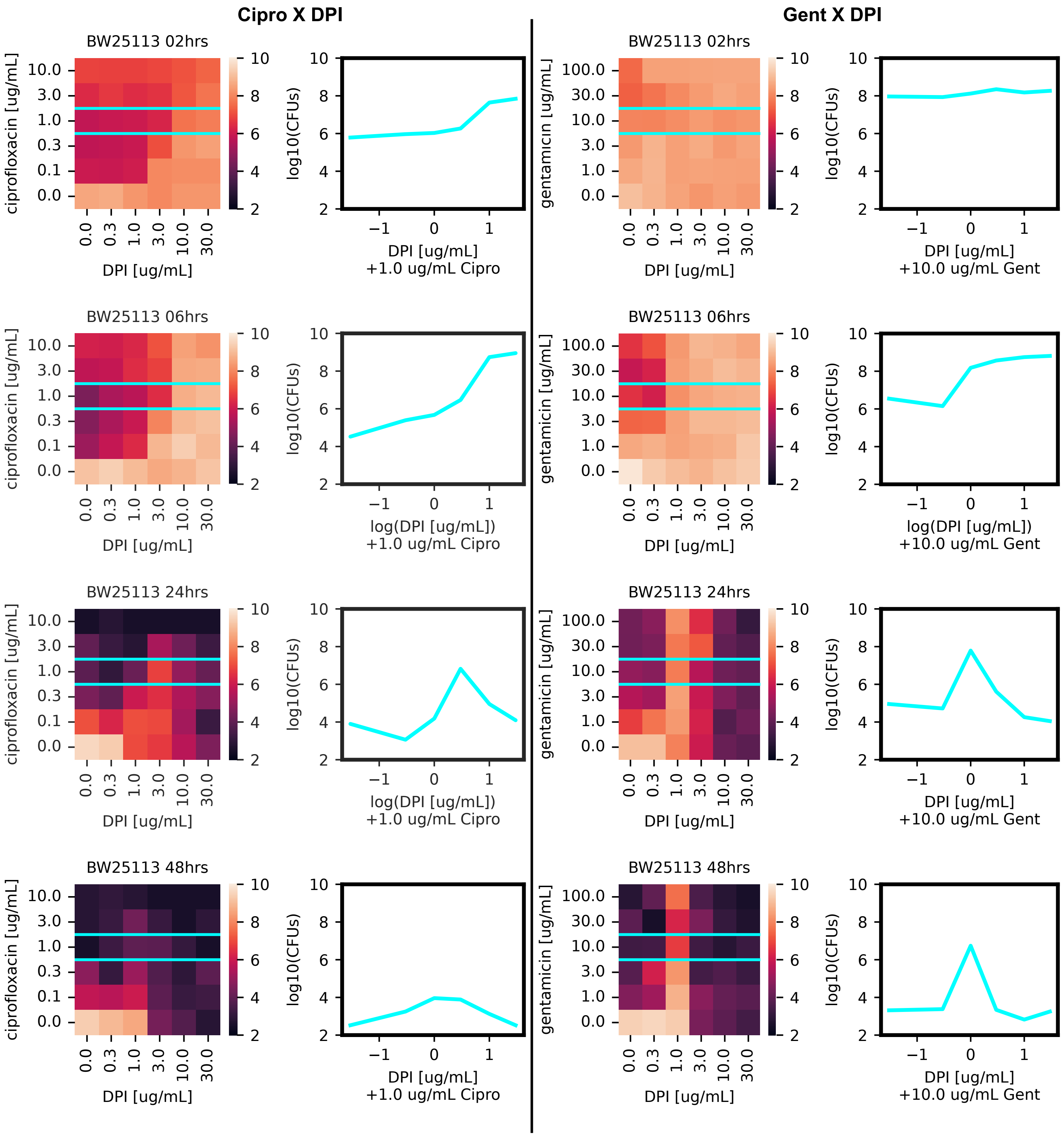

Figure S9: **GVA temporal checkerboard of DPI crossed with either ciprofloxacin (left panels) or gentamicin (right panels) against *E. coli*.** Treatment time increases down the rows. Each square in the heatmap was the mean of duplicate conditions. Colorbar correspond to the measured log10(CFUs/mL) for each combination. Left panel shows line trace (cyan) for the DPI dose response at 1 μg/mL ciprofloxacin or 10 μg/mL gentamicin.

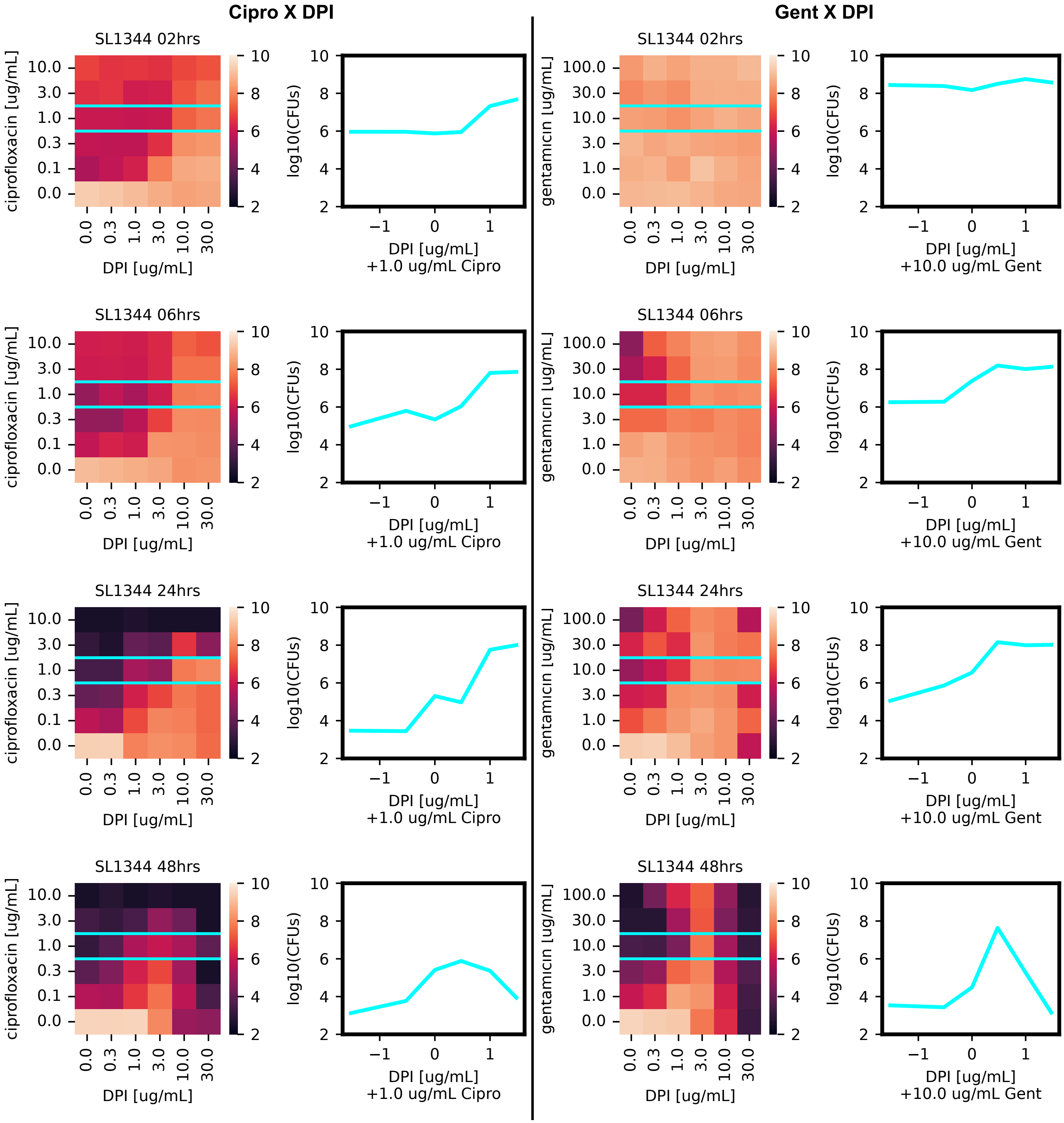

Figure S10: **GVA temporal checkerboard of DPI crossed with either ciprofloxacin (left panels) or gentamicin (right panels) against *S. typhimurium*.**

4 Derivation of the axial probability density function for a cone.

Assuming single cells are well mixed before being suspended and cast into a 3D cone, the probability of a colony forming at distance x from the origin is proportional to the percent of the total volume (V) comprised by the infinitesimal volume (dV) at x. dV is defined as

$$dV = \pi r'(x)^2 * dx \tag{1}$$

where $r'(x)$ is the radius of the circle at x (Extended Data Fig. 1a, cyan circle). Based on the geometry in Extended Data Fig. 1a (right panel), we find

$$r'(x) = \frac{r}{h} * x \tag{2}$$

where r is the radius of the cone's base and h is the height of the cone.

The probability density function (PDF) for this geometry can be solved for by

$$C * \int_0^h \frac{\pi r^2}{h^2} x^2 dx = 1 \tag{3}$$

where C is the normalization constant and is equal to the inverse of the the volume V (i.e. $C = \frac{3}{\pi h r^2}$) This leads to the following PDF (Extended Data Fig. 1b)

$$PDF(x) = \frac{3 * x^2}{h^3} \tag{4}$$

The associated Cumulative Distribution Function (CDF) can be found from the integral (Extended Data Fig. 1c)

$$CDF(x) = \frac{x^3}{h^3} \tag{5}$$

We can observe here that regardless of the base shape of the cone or pyramid, as long as it is axially symmetric, this PDF holds (Extended Data Fig. 1d) as a result of the specific geometry of dV canceling out of the PDF due to the normalization constant.

Following the same derivation, the PDF of a cylinder is found to be a constant $\frac{1}{h}$ and the PDF of a 2D wedge is $\frac{2*x}{h^2}$ (Fig. 1b). Because of the exponent on x, the PDF of the cone gives the largest dynamic range in the probability (Fig. 1b).

The CDF measures the likelihood of having found a colony as function of x if only a single colony is in the cone (Fig. 1b). The PDF is the probability of finding a colony at any point x for only a single colony in the cone. Therefore, there are two equivalent ways of calculating the number of CFUs using either the PDF or the CDF (Extended Data Fig. 1e).

With the PDF we can estimate the number of CFUs/mL using the equation

$$CFUs/mL = \frac{N(x)|x_1 \leq x < x_2}{V * \int_{x_1}^{x_2} PDF(x)dx} \tag{6}$$

where x_1 and x_2 is the position of the first and last colony and V is the cone volume.

With the CDF,

$$CFUs/mL = \frac{N(x)|x < x_2}{V * CDF(x_2)} \tag{7}$$

In practice, we find using the PDF estimator to be more convenient because it does not depend on identifying the first colony from the tip, but mathematically these are equivalent.

References

1. Levin-Reisman, I. *et al.* Antibiotic tolerance facilitates the evolution of resistance. *Science* **355,** 826–830 (Feb. 2017).
2. Zheng, E. J., Stokes, J. M. & Collins, J. J. Eradicating Bacterial Persisters with Combinations of Strongly and Weakly Metabolism-Dependent Antibiotics. *Cell chemical biology* **27,** 1544–1552 (Dec. 2020).
3. Leslie, D. J. *et al.* Nutritional Control of DNA Replication Initiation through the Proteolysis and Regulated Translation of DnaA. *PLoS Genetics* **11** (July 2015).
4. Zeiler, H. J. & Voigt, W. H. Efficacy of ciprofloxacin in stationary-phase bacteria in vivo. *The American Journal of Medicine* **82,** 87–90 (Apr. 1987).
5. Levin, B. R. & Rozen, D. E. Non-inherited antibiotic resistance. *Nature Reviews Microbiology* **4,** 556–562 (July 2006).
6. SW, L., EJ, F. & JA, E. Mode of Action of Penicillin: I. Bacterial Growth and Penicillin Activity-Staphylococcus aureus FDA. *Journal of bacteriology* **48,** 1036 (Nov. 1944).
7. Finkel, S. E. Long-term survival during stationary phase: evolution and the GASP phenotype. *Nature Reviews Microbiology* **4,** 113–120 (Feb. 2006).
8. Aldieri, E. *et al.* Classical inhibitors of NOX NAD(P)H oxidases are not specific. *Current drug metabolism* **9,** 686–696 (Oct. 2008).
9. Pandey, M. *et al.* Diphenyleneiodonium chloride (DPIC) displays broad-spectrum bactericidal activity. *Scientific Reports* **7,** 1–8 (Sept. 2017).
10. Jung, B., Li, T., Ji, S. & Lee, J. Efficacy of Diphenyleneiodonium Chloride (DPIC) Against Diverse Plant Pathogens. *Mycobiology* **47,** 105 (2019).
11. Dwyer, D. J., Kohanski, M. A. & Collins, J. J. Role of Reactive Oxygen Species in Antibiotic Action and Resistance. *Current opinion in microbiology* **12,** 482 (Oct. 2009).
12. Kohanski, M. A., Dwyer, D. J., Hayete, B., Lawrence, C. A. & Collins, J. J. A Common Mechanism of Cellular Death Induced by Bactericidal Antibiotics. *Cell* **130** (2007).
13. Hong, Y., Zeng, J., Wang, X., Drlica, K. & Zhao, X. Post-stress bacterial cell death mediated by reactive oxygen species. *Proceedings of the National Academy of Sciences of the United States of America* **116,** 10064–10071 (May 2019).
14. Choi, H., Yang, Z. & Weisshaar, J. C. Single-cell, real-time detection of oxidative stress induced in escherichia coli by the antimicrobial peptide CM15. *Proceedings of the National Academy of Sciences of the United States of America* **112,** E303–E310 (Jan. 2015).

15. Schoemaker, J. M., Gayda, R. C. & Markovitz, A. Regulation of cell division in Escherichia coli: SOS induction and cellular location of the SulA protein, a key to lon-associated filamentation and death. *Journal of Bacteriology* **158,** 551–561 (1984).

16. Baharoglu, Z. & Mazel, D. SOS, the formidable strategy of bacteria against aggressions. *FEMS Microbiology Reviews* **38,** 1126–1145 (Nov. 2014).

17. Zaslaver, A. *et al.* A comprehensive library of fluorescent transcriptional reporters for Escherichia coli. *Nature Methods* **3,** 623–628 (Aug. 2006).

18. Podlesek, Z. & Žgur Bertok, D. The DNA Damage Inducible SOS Response Is a Key Player in the Generation of Bacterial Persister Cells and Population Wide Tolerance. *Frontiers in Microbiology* **11,** 1785 (Aug. 2020).

19. Juillan-Binard, C. *et al.* A Two-component NADPH Oxidase (NOX)-like System in Bacteria Is Involved in the Electron Transfer Chain to the Methionine Sulfoxide Reductase MsrP. *Journal of Biological Chemistry* **292,** 2485–2494 (Feb. 2017).

20. Hocquet, D. & Bertrand, X. Metronidazole increases the emergence of ciprofloxacin- and amikacin-resistant Pseudomonas aeruginosa by inducing the SOS response. *Journal of Antimicrobial Chemotherapy* **69,** 852–854 (Mar. 2014).
