# Supplementary material for: A high-throughput and low-waste viability assay for microbes
Source: Nat Microbiol. 2023 Nov 2;8(12):2304–14. doi: 10.1038/s41564-023-01513-9 (PMC10686820; doi:10.1038/s41564-023-01513-9)
Supplement: Supplementary file 2 — Reporting Summary [file 41564_2023_1513_MOESM2_ESM.pdf]

nature portfolio

Corresponding author(s): Christian T. Meyer, Anushree Chatterjee, Joel M. Kralj

Last updated by author(s): Sep 18, 2023

Reporting Summary

Nature Portfolio wishes to improve the reproducibility of the work that we publish. This form provides structure for consistency and transparency in reporting. For further information on Nature Portfolio policies, see our Editorial Policies and the Editorial Policy Checklist.

Statistics

For all statistical analyses, confirm that the following items are present in the figure legend, table legend, main text, or Methods section.

n/a	Confirmed	
☐	☒	The exact sample size (n) for each experimental group/condition, given as a discrete number and unit of measurement
☐	☒	A statement on whether measurements were taken from distinct samples or whether the same sample was measured repeatedly
☐	☒	The statistical test(s) used AND whether they are one- or two-sided<br>*Only common tests should be described solely by name; describe more complex techniques in the Methods section.*
☒	☐	A description of all covariates tested
☐	☒	A description of any assumptions or corrections, such as tests of normality and adjustment for multiple comparisons
☐	☒	A full description of the statistical parameters including central tendency (e.g. means) or other basic estimates (e.g. regression coefficient) AND variation (e.g. standard deviation) or associated estimates of uncertainty (e.g. confidence intervals)
☐	☒	For null hypothesis testing, the test statistic (e.g. F, t, r) with confidence intervals, effect sizes, degrees of freedom and P value noted<br>*Give P values as exact values whenever suitable.*
☒	☐	For Bayesian analysis, information on the choice of priors and Markov chain Monte Carlo settings
☒	☐	For hierarchical and complex designs, identification of the appropriate level for tests and full reporting of outcomes
☐	☒	Estimates of effect sizes (e.g. Cohen's d, Pearson's r), indicating how they were calculated

Our web collection on statistics for biologists contains articles on many of the points above.

Software and code

Policy information about availability of computer code

Data collection	Custom software for data acquisition is made available in the manuscript (Arduino 2.2.1, digiCamControl v2.1, MATLAB 2022a). This code is available from the following DOI: https://doi.org/10.5281/zenodo.8342460. All use is governed by the associated license file included with the download. For image aquisition NIKON ELEMENTS software was used.
Data analysis	All data analysis was run in MATLAB 2022a or python 3.7 scripts.

For manuscripts utilizing custom algorithms or software that are central to the research but not yet described in published literature, software must be made available to editors and reviewers. We strongly encourage code deposition in a community repository (e.g. GitHub). See the Nature Portfolio guidelines for submitting code & software for further information.

Data

Policy information about availability of data

All manuscripts must include a data availability statement. This statement should provide the following information, where applicable:

- Accession codes, unique identifiers, or web links for publicly available datasets
- A description of any restrictions on data availability
- For clinical datasets or third party data, please ensure that the statement adheres to our policy

The Enzo bioactive screen viability data is published on Zenodo with the following DOI: https:
530 //doi.org/10.5281/zenodo.7986816. All data needed to evaluate the conclusions in the paper are present in the paper and/or in the Supplementary Materials.

Research involving human participants, their data, or biological material

Policy information about studies with human participants or human data. See also policy information about sex, gender (identity/presentation), and sexual orientation and race, ethnicity and racism.

Reporting on sex and gender	NA
Reporting on race, ethnicity, or other socially relevant groupings	NA
Population characteristics	NA
Recruitment	NA
Ethics oversight	NA

Note that full information on the approval of the study protocol must also be provided in the manuscript.

Field-specific reporting

Please select the one below that is the best fit for your research. If you are not sure, read the appropriate sections before making your selection.

☒ Life sciences ☐ Behavioural & social sciences ☐ Ecological, evolutionary & environmental sciences

For a reference copy of the document with all sections, see nature.com/documents/nr-reporting-summary-flat.pdf

Life sciences study design

All studies must disclose on these points even when the disclosure is negative.

Sample size	No sample size pre-tests were calculated. Sample sizes were determined by experimental constraints on data acquisition. Sample size were sufficient deemed sufficient based on the measured noise between replicates.
Data exclusions	No data excluded from the analysis.
Replication	The dilution series of E. coli using GVA has been replicated by 3 different people including members of another lab group.
Randomization	No randomization required. No covariates were tested in the study and are not relevant to the proof of the GVA technique.
Blinding	No blinding was done during this study. Blinding was not done as it was not required to verify the performance of the technique which was directly compared to the gold standard Drop CFU approach.

Reporting for specific materials, systems and methods

We require information from authors about some types of materials, experimental systems and methods used in many studies. Here, indicate whether each material, system or method listed is relevant to your study. If you are not sure if a list item applies to your research, read the appropriate section before selecting a response.

Materials & experimental systems

n/a	Involved in the study	
☒	☐	Antibodies
☐	☒	Eukaryotic cell lines
☒	☐	Palaeontology and archaeology
☒	☐	Animals and other organisms
☒	☐	Clinical data
☒	☐	Dual use research of concern
☒	☐	Plants

Methods

n/a	Involved in the study	
☒	☐	ChIP-seq
☒	☐	Flow cytometry
☒	☐	MRI-based neuroimaging

Eukaryotic cell lines

Policy information about cell lines and Sex and Gender in Research

Cell line source(s)	BY4741 (S. cerevisiae) was a gift from the Parker lab at University of Colorado Boulder.
Authentication	None
Mycoplasma contamination	None
Commonly misidentified lines (See ICLAC register)	None
