# Supplementary material for: A high-throughput and low-waste viability assay for microbes
Source: Nat Microbiol. 2023 Nov 2;8(12):2304–14. doi: 10.1038/s41564-023-01513-9 (PMC10686820; doi:10.1038/s41564-023-01513-9)
Supplement: Supplementary file 5 — Detailed protocol for the GVA assay. [file 41564_2023_1513_MOESM5_ESM.pdf]

GVA embedding protocol for 96 well plate using pipette tips

Materials:

Agarose (Bacto-agar, Low melt agar, etc.)

Growth medium (*e.g.,* LB)

1 full p200 tip box sterilized

1 empty p200 tip box sterilized

1 old tip box base without lid used for as an ice bucket

1 multichannel pipette

1 reservoir

1 water/bead bath (set to between 42-50°C for Bacto-agar, 37°C for Low melt agar).

1 incubator

96 well plate (round bottom preferred but flat bottom works)

TTC (optional, bacterial colony stain for increasing colony contrast)

Protocol:

1. Start prep work 1 hour prior to embedding a sample.
2. Add 0.66 g agarose into 100mL of growth medium (0.66% w/v solution) in a sterilized bottle with a cap.
    a. Notes: The subsequent mixing with the sample results in a final concentration of 0.5% agarose which has an average pore size of 600nm. Increasing the agarose concentration decreases the pore size and accelerates the speed the gel sets in the tip. See this paper (Figure 3) for pore size as a function of agarose concentration: link (Narayanan J et al, J. Phys. Conf. Ser. 2006).
3. Adjust the cap tightness to allow steam to escape before microwaving the agarose media mixture on low power till the agarose is *completely* melted. Swirl the bottle to make sure no agarose remains. Place the melted agarose in a water/bead bath and allow to equilibrate for 1 hour.
    a. Notes: BE CAREFUL MICROWAVING AGAROSE! Alternatively, autoclave the media and place it in the warm bath where it will keep for a few days.
    b. Be sure the water bath level is well above the level of the melted agarose in the bottle.
4. (Optional) Thaw TTC stock (25 mg/mL 1000X in water).
5. After 1 hour, make an ice bath by filling an old tip box with ice. Then add enough water that the ice can move freely.
6. Pipette 50 µL of each treated condition ($<10^7$ CFUs/mL) into separate wells in a 96 well plate.
    a. If your sample has $>10^7$ CFUs/mL, dilute the sample before running. One method for stationary phase cells ($\sim10^9$) is to place 50 µL of media in all wells of a 96 well plate and then use a pin tool to transfer 500 nL from the original sample plate.
7. Remove agarose from the heat bath. Optionally add 100 µL TTC stock (final concentration 25 µg/mL) to agarose.
8. Pour out all the agarose into the reservoir. 100 mL is more than needed for one 96 well plate, but the large volume keeps the agarose from gelling too fast.
    a. To further slow down the agarose gelling, place the reservoir in a warm bead bath held at around 50°C.
    b. Increasing the concentration of agarose will speed up gelling times.
9. Working quickly, use a 12 channel pipette to mix 150 µL liquid media-agarose into the wells containing the 50 µL of sample. Mix 2-5x slowly.
    a. The limit of detection (LOD) of a 50 µL sample mixed with 150 µL is ~26.66 CFUs/mL (4x dilution into agarose * 1000/150). To further decrease the LOD change the ratio of sample to melted agarose adjusting the agarose concentration accordingly.

10. Aspirate 150 µL slowly after mixing, being sure to keep the tips near the bottom of the well but not pressing down to prevent the formation of bubbles in the tip and submerge tips in ice bath for ~6 sec.
    a. Notes: Avoid bubbles by reducing speed and positioning pipette tips in the bottom of the well.
    b. Some media's salt concentration (*e.g.,* YEPD) results in slower gelling times. Optimize for your system.
    c. When removing from the ice bath, angle the tips and gently scrape them along the ice to remove any globs that might be clinging to the side.
    d. If using low melt agarose try doubling the time in the ice bath.
11. Eject tips into an empty tip box and repeat for remaining wells.
    a. If using an electronic pipette, be sure to turn off the function which auto zeros the pistons position once the tips are ejected as this can lead to premature ejection of the semi solid agarose.
12. Allow tips to set at room temperature for half hour before placing them in an incubator overnight with a beaker of water for increasing humidity.
13. Measure the colony positions the following day. Tips can be kept at room temperature for several days with no appreciable change in the colony size.
